# Dose-response effects of multiple *Ascaris suum* exposures and their impact on lung protection during larval ascariasis

**DOI:** 10.1371/journal.pntd.0012678

**Published:** 2024-12-02

**Authors:** Chiara Cássia Oliveira Amorim, Denise Silva Nogueira, Ana Clara Gazzinelli-Guimarães, Thais Leal-Silva, Fernando Sérgio Barbosa, Fabrício Marcus Silva Oliveira, Lucas Rocha Kraemer, Raquel Martins de Almeida, Jorge Lucas Nascimento Souza, Luisa Mourão Dias Magalhães, Remo Castro Russo, Marcelo Vidigal Caliari, Soraya Gaze, Lilian Lacerda Bueno, Ricardo Toshio Fujiwara

**Affiliations:** 1 Laboratory of Immunobiology and Parasite Control, Department of Parasitology, Institute of Biological Sciences, Universidade Federal de Minas Gerais, Minas Gerais, Brazil; 2 Federal District Health Department (SES/DF), Distrito Federal, Brazil; 3 Laboratory of Interactions in ImmunoParasitology, Department of Parasitology, Institute of Biological Sciences, Universidade Federal de Minas Gerais, Minas Gerais, Brazil; 4 Laboratory of Pulmonary Immunology and Mechanics, Department of Physiology and Biophysics, Institute of Biological Sciences, Universidade Federal de Minas Gerais, Minas Gerais, Brazil; 5 Department of General Pathology, Institute of Biological Sciences, Universidade Federal de Minas Gerais, Minas Gerais, Brazil; 6 Cellular and Molecular Immunology Group, René Rachou Institute, Oswaldo Cruz Foundation–FIOCRUZ, Minas Gerais, Brazil; National Institute of Health, INDIA

## Abstract

**Background:**

Human ascariasis is the most prevalent geohelminthiasis worldwide, affecting approximately 446 million individuals. In regions with endemic prevalence, the majority of infected adults are frequently exposed to the parasite and tend to have a low parasite load. Further studies are necessary to provide more evidence on the dynamics of infection and to elucidate the possible mechanisms involved in regulating protection, especially during the acute phase, also known as larval ascariasis. The aim of this study is to compare the impact of lung function between single and multiple infections in a murine model.

**Methods:**

We infected BALB/c mice considering the frequency of exposures: single-exposure—SI; twice-exposures—RE 2x and thrice-exposures—RE 3x, and considering the doses of infection: 25 eggs—RE 25; 250 eggs—RE 250 and 2,500 eggs—RE 2500, followed by infection challenge with 2,500 eggs. From this, we evaluated: parasite burden in lungs, cellular and humoral response, histopathological and physiological alterations in lungs.

**Results:**

The main results showed a reduction of parasite burden in the reinfected groups compared to the single-infected group, with protection increasing with higher exposure and dose. Furthermore, the RE 250 group exhibited a decrease of parasite burden close to RE 2500, but with less tissue damage, displaying the most favorable prognosis among the reinfected groups.

**Conclusion:**

Our research indicates a dose-dependent relationship between antibody production and the intensity of the immune response required to regulate the parasite burden.

## Author summary

Human ascariasis is a common and neglected disease and is the most prevalent geohelminthiasis worldwide. In certain regions considered endemic, recurrent infections are frequent. This is mainly due to inadequate sanitation infrastructure and ineffective health education. This study aims to better understand the dynamics of *Ascaris* infection and thus elucidate the possible mechanisms involved in the regulation of protection, especially during the acute phase, also known as larval ascariasis. To this end, we infected BALB/c mice with different frequencies of exposure and different doses of infection. We observed a reduction in larval recovery in the re-infected groups compared to the single-infection group. This protection increased with increasing exposure frequency and dose. In addition, we found that the production of antibodies and the intensity of the immune response appeared to be related to the regulation of parasite burden.

## Introduction

Human ascariasis is the most common intestinal infection caused by helminths among neglected tropical diseases. It is important to public health, mainly due to its socioeconomic impact on endemic areas [1]. In 2019, the global estimate of population loss caused by ascariasis was 754,000 disability-adjusted life years, and current data estimates that approximately 446 million people worldwide are infected with *Ascaris* spp. [2], mainly school-age children residing in developing countries. The precariousness of the basic sanitation system and the inefficiency of health education efforts in endemic areas contribute to the maintenance of geohelminthiasis in these regions, resulting in high reinfection rates even after specific treatment [3]. Experimental and molecular studies have demonstrated the possible existence of cross-transmission, indicating that humans can be infected by *Ascaris suum* [4–6] and pigs can harbor *A*. *lumbricoides* [7]. This means that pigs may serve as a potential reservoir for human zoonotic infections. Prophylactic interventions against human ascariasis are insufficient, allowing the maintenance of the parasite cycle in the environment.

In areas with high endemicity, most individuals are often exposed multiple times to the parasite, and typically have a low parasite burden. This suggests that the host is capable of developing a protective immune response against the infection, as demonstrated in an experimental model of ascariasis [8]. It is believed that an extensive systemic, airway, and lung inflammatory response is initiated to regulate and control larval migration. In an attempt to elucidate immunological pathways and pathophysiological aspects, many studies on experimental models use a high standard dose of 2,500 fully embryonated eggs for infection [8–11]. Nevertheless, exposure to the worm in natural settings typically involves repeated low doses, leading to the development of acquired or concomitant immunity [12]. Currently, a standardized model that effectively replicates natural infection is lacking.

Further assessments are necessary to enhance our understanding of the biology underlying the interaction between *Ascaris* and the host. Despite the evidence from studies involving multiple exposures, there is a need to elucidate the immunological mechanisms and protective pathways that regulate parasite burden and minimize tissue damage in larval ascariasis. From this perspective, it is crucial to use models that better mimic natural infection conditions to compare different approaches to multiple infections and doses of eggs used in experimental infections. In the current study, we assess the effects of *Ascaris suum* infection in BALB/c mice by investigating exposure frequency and infection doses during the pulmonary phase of larval ascariasis. This exploration aims to comprehensively understand the parasite-host relationship and contribute valuable insights to the development of prophylactic measures and vaccines against *Ascaris* spp. infection

## Materials and methods

### Ethics statement

All research activities involving animal models were conducted following to the Brazilian College of Animal Experimentation (COBEA) and approved by the Comissão de Ética no Uso de Animais (CEUA)–“Ethics Committee in the Use of Animals” of the UFMG, as certified by Protocol No. 221/2020. All efforts were made to minimize animal suffering.

### Parasites

Adult *A*. *suum* worms were harvested from pigs at a Brazilian slaughterhouse (Belo Horizonte, Minas Gerais, Brazil). Eggs were isolated from the uteri of female worms by gentle mechanical maceration and further purified by use of cell strainers (70 μm). Isolated eggs were incubated with 0.2 M H_2_SO_4_ for embryonation, as described by Boes and colleagues, (1998) [13]. After the 100^th^ day of culture, corresponding to the peak of larval infectivity [9], fully embryonated eggs were used in experimental infections.

### Experimental design

Eight-week-old female BALB/c mice (n = 90) from the Central Animal Facility of the Universidade Federal de Minas Gerais (UFMG), Brazil were used.

**Fig 1:**
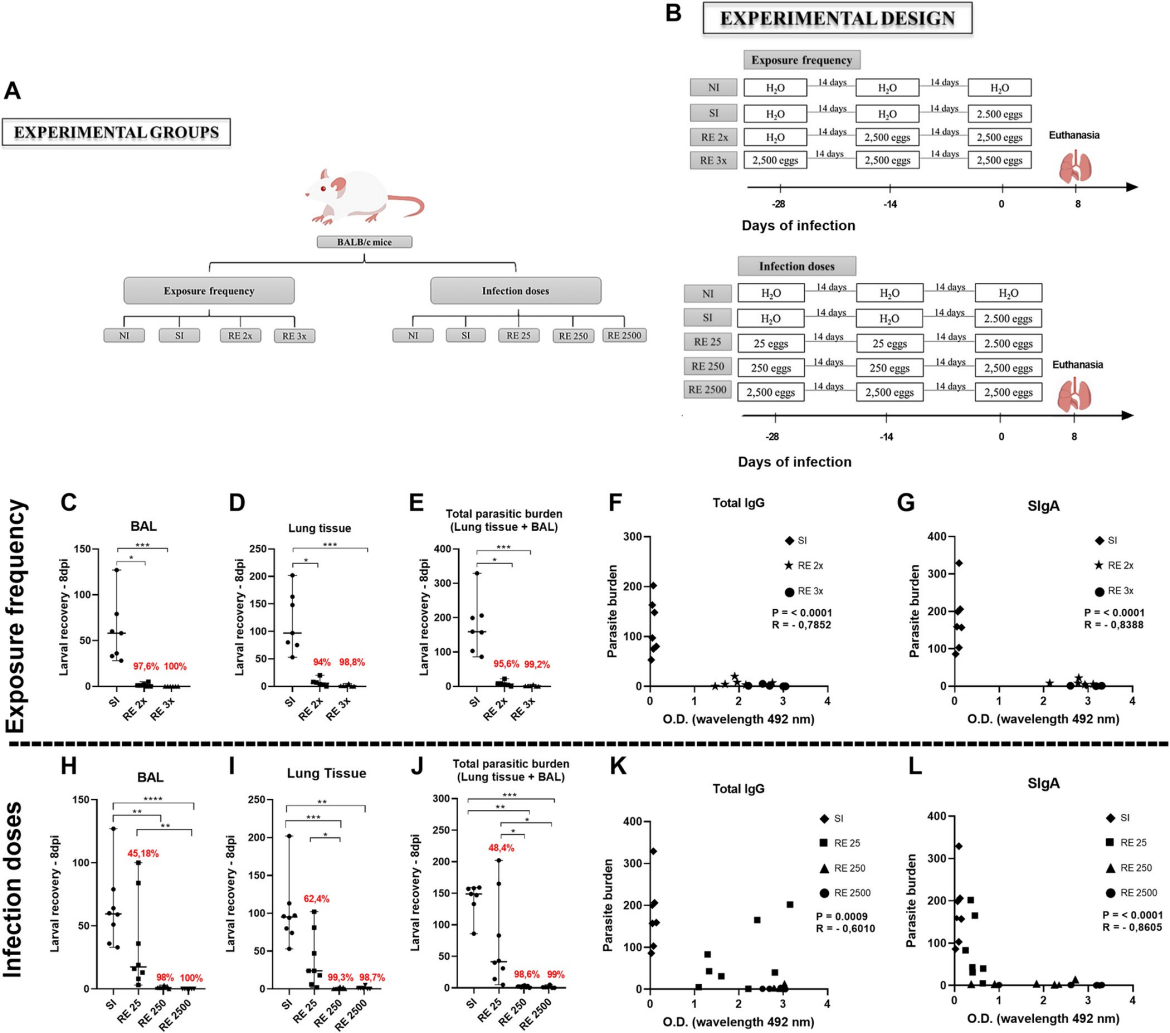
Experimental design and parasite burden and correlation with animal antibody production as a function of exposure frequency and infection dose. (A and B) Female BALB/c mice were divided according to exposure frequency and infection doses into the following groups, No-infected (NI), Single-Infected (SI) Reinfected 2x (RE 2x), Reinfected 3x (RE 3x), Reinfected with 25 eggs (RE 25), Reinfected with 250 eggs (RE 250) and Reinfected with 2500 (RE 2500). Larvae were recovered and quantified after 8 days of infection in BALB/c mice using the modified Baermann method. (C and H) Number of larvae recovered from bronchoalveolar lavage; (D and I) Number of larvae recovered from lung tissue; (E and J) Total number of larvae recovered (BAL + tissue). Correlation between antibody production and parasite burden is shown in (F and K) IgG levels and parasite burden; (G and L) SIgA levels and parasite burden. Significant differences between groups are indicated by (*). Where *p<0.05, **p<0.01, ***p<0.001, ****p<0.0001. Results are presented in graphs with a median and 95% CI. Figure created by the authors.

For the necessary analyses, mice were divided into six different groups, namely: No-infected (NI), which received only filtered water at all times of infection; Single-infected (SI) who received two doses of filtered water and one dose containing 2,500 fully embryonated *A*. *suum* eggs at the last time of infection; Reinfected twice (RE 2x) who received one dose of filtered water in the once of infection and two doses containing 2,500 fully embryonated eggs in the second and last time of infection; Reinfected three times or Reinfected with 2,500 eggs (RE 3x or RE 2500) who received three doses of 2,500 fully embryonated *A*. *suum* eggs at all times of infection; Reinfected with 250 eggs (RE 250) who received two doses containing 250 fully embryonated *A*. *suum* eggs in the first two times of infection and a challenge dose containing 2,500 fully embryonated eggs in the last time; Reinfected with 25 eggs (RE 25) who received two doses containing 25 fully embryonated *A*. *suum* eggs and a challenge dose containing 2,500 fully embryonated eggs (Fig 1A and 1B).

The groups were separated in such a way as to make it possible to assess the impacts generated by different exposure frequencies and different infection doses on parasite control. At the peak of larval migration in the lungs (eighth day after infection - 8dpi), as previously described [9], the animals were euthanized to perform parasitological, immunological, physiological and histopathological evaluations.

### Experimental infection and parasitological analysis

The preparation of eggs for infections was carried out according to Gazzinelli-Guimarães et al., (2013) [9]. After the thorough washing sequence, eggs were quantified for the respective infections of the different groups. Therefore, the sediment was suspended in 10 mL of filtered water and three aliquots of 10 μL were collected from the suspension and used to count larval eggs. The value obtained was used to calculate the average number of fully larvated eggs per μL and to determine the necessary dilution to get a suspension containing 2,500 eggs in 0.2 mL, 250 eggs in 0.2 mL and 25 eggs in 0.2 mL.

After preparing the eggs, the infection was performed intragastrically according to the protocol described by Lewis et al., (2006) [14], in which, with the aid of a gavage needle, approximately 100 μL of a suspension containing the eggs were inoculated., according to the group being infected, followed by 100 μL of water to remove the remaining eggs in the syringe and needle. Animals in the no-infection group were administered 200 μL of filtered water (Fig 1B) Parasite burden was evaluated by measuring the larvae recovery from the lungs. Tissues were collected, sliced with scissors and placed in a Baermann apparatus for 4 hours in the presence of PBS at 37°C. The recovered larvae were then fixed (1% formaldehyde in PBS) and counted under an optical microscope at 10x magnification.

### Pulmonary cytokine profile

The right lung (quadrilobed) was collected to evaluate the cytokine profile in lung tissue. Afterward, 100 mg of lung tissue was homogenized in 1 mL of PBS (0.4 M NaCl and 10 mM NaPO4) supplemented with protease inhibitors (0.1 mM phenylmethylsulfonyl fluoride, 0.1 mM sodium chloride benzethonium, 10 mM EDTA and 20 KI aprotinin A) and 0.05% Tween 20. The homogenates were centrifuged at 8,000 x g for 10 minutes at 4°C and the supernatants were collected and stored at -80°C, and subsequently used to measure cytokines. The determination of IL-5, IL-13, IFN-γ and TNF-α cytokine production was performed using an ELISA kit (R&D Systems, USA) according to the manufacturer’s instructions. The absorbance was determined by a VersaMax ELISA microplate reader (Molecular Devices, USA) at a wavelength of 492 nm.

### Eosinophil peroxidase, neutrophil myeloperoxidase, and macrophage N-Acetyl-beta-D-Glucosamidase assays

The indirect activity of eosinophils, neutrophils and macrophages was evaluated by the concentration of eosinophil peroxidase (EPO), neutrophil myeloperoxidase (MPO), and N-acetylglucosaminidase (NAG) in lung homogenates performed according to the previously described method [8,15]. After tissue homogenization (Power Gen 125—Fisher Scientific, USA), the lung homogenate was centrifuged at 1,500 x g for 10 minutes at 4°C and the resulting pellet was then used to determine the activity of the cells in question. The absorbance was expressed by a VersaMax ELISA Microplate Reader (Molecular Devices, USA) according to each assay and the results were expressed as optical densities (O.D.).

### Bronchoalveolar lavage

To evaluate lung damage and airway inflammation by analysis of bronchoalveolar lavage (BAL), the mice were anesthetized and a 1.7 mm catheter was inserted into the animal’s trachea. 1 mL of PBS was used twice for perfusion and aspiration to assess leukocyte infiltration into the bronchoalveolar compartment. Bronchoalveolar lavage was filtered through 70 μm cell filters (BD, USA) to retrieve and quantify *A*. *suum* larvae numbers in BAL fluid. The material was centrifuged at 300 x g for 10 minutes at 4°C and the pellet was used for the experiment of the total number of leukocytes and differentiation of macrophages, lymphocytes, eosinophils, and neutrophils by optical microscopy at 100x magnification. The supernatant was used to quantify the amount of total protein and hemoglobin content. Samples from the NI group were used as controls.

The BCA Protein Assay kit (Thermo Scientific, USA) determined the total protein quantification in BAL to measure the protein leakage into the airways, as previously described [16]. The results were expressed as μg of total protein per mL of BAL. The extent of the alveolar hemorrhage was assessed by the amount of free hemoglobin (Hb) detected in the BAL supernatant using the Drabkin method, as previously described [17]. The hemoglobin concentration in the samples was determined spectrophotometrically by measuring absorbance at 540 nm and interpolation from a standard hemoglobin curve, starting at 1 mg/mL. Hemoglobin content was expressed as μg of Hb per mL of BAL.

### Detection of serum IgG production, specific total and subclasses, and SIgA production in BAL

ELISA assays were performed using serum from animals from different groups. ELISA plates (Greiner-Bio-One, USA) were sensitized with 100 μL of crude adult worm antigen at a concentration of 10 μg per well and left overnight at 4°C. The next day, after sensitization, the plates were washed 5 times with the washing solution (PBS-0.05% Tween20) and then the plate was blocked with 250 μL of PBS plus 3% BSA for 1 hour at 37°C. After blocking, all the solution in the wells was removed, and 100 μL of sera from the animals in the experimental groups diluted 1:1,000 in PBS with 3% BSA were added to the wells and incubated at 4°C overnight. The plates, on the following day, were washed 5 times with the washing solution (PBS-0.05% Tween20) and 100 μL of anti-mouse IgG antibody conjugated with peroxidase diluted 1:2,000 in PBS-BSA 3% was added. After incubation at 37°C for 1 hour, the plates were rewashed 5 times with the washing solution. Then 100μL of the developing solution was added, containing 0.1 M citric acid, 0.2 M Na_2_PO_4_, 0.05% OPD (o-phenylenediamine dihydrochloride) and 0.1% H_2_O_2_. The plates were incubated at 37°C away from light for 20 minutes with the developing solution and the reaction was stopped by adding 50μL of 2M H_2_SO_4_. Optical density was read on a VersaMax ELISA reader (MolecularDevices, USA) at 492nm. All assays were performed in duplicates.

In the standard ELISA for IgG subclasses, the protocol performed was the same as described above, using the respective secondary anti-mouse IgG1, anti-mouse IgG2a, anti-mouse IgG2b and anti-mouse IgG3 antibodies all at a 1:1,000 dilution in PBS-BSA 3%. Animal sera were diluted 1:500 for IgG1 and IgG3 and 1:100 for IgG2a and IgG2b. To determine of SIgA, the BAL sample was used without dilution and the secondary antibody at a 1:500 dilution in PBS-BSA 3%.

### Histopathological analysis

The left lung was collected on the eighth-day post-infection and fixed in 10% formalin solution for two days, then gradually dehydrated in ethanol, diaphanized in xylol, and embedded in paraffin blocks that were cut at a thickness of 4 μm and fixed on microscopy slides. Slides with lung tissue were stained with hematoxylin and eosin (H&E) for the tissue damage, considering the intensity of inflammation and hemorrhage.

The slides were examined under a light field optical microscope coupled to a digital system image capture camera (Motic 2.0). Two analyses were used for the histopathological analysis in the animals’ lungs after the larvae migration through the organ. For analysis of the degree of airway inflammation, peribronchial inflammation, perivascular inflammation, parenchymal inflammation, and presence of hemorrhage in the lungs, a semiquantitative analysis was performed following the methodology previously described by Gazzinelli-Guimarães et al., (2018) [18]. S1 Fig shows the histopathology scoring system for mice lungs. In addition, a histopathological description was also carried out to evaluate the lesions caused by larval migration (at 8 dpi) in mice’s lung parenchyma, considering cell infiltration, inflammation, and exudative phenomena.

### Assessment of respiratory mechanics

The assessment of lung function was performed as previously described [8,18,19]. Briefly, mice were anesthetized with a subcutaneous injection of ketamine and xylazine (8.5 mg/kg xylazine and 130 mg/kg ketamine) to maintain spontaneous breathing. Then, the animals were tracheostomized, placed in a body plethysmograph and connected to a computer-controlled ventilator (Forced Pulmonary Maneuver System, Buxco Research Systems, Wilmington, North Carolina, USA). This lab setup, designed specifically for use in mice, has only a cannula volume (death space) of 0.8 mL and provides three semi-automatically maneuvers: Boyle’s Law FRC, quasi-static pressure-volume, and fast flow volume. First, pressure-controlled ventilation imposed an average respiratory rate of 160 breaths/min on the anesthetized animal until a regular breathing pattern and complete expiration with each breath cycle was achieved. Under mechanical respiration, the RC Resistance and Compliance test determined Dynamic Compliance (Cdyn) and Pulmonary Resistance (Rl). Suboptimal maneuvers were rejected, and for each test in each mouse, at least three acceptable maneuvers were performed to obtain a reliable average for all numerical parameters. At the end of the experiment, the mice were euthanized, and the organs were collected for further analysis.

### Statistical analysis

The GraphPad Prism 8 program (GraphPad Inc, USA) was used to analyze the data generated in this work. First, the ROUT test was performed to detect the presence of possible outliers in the samples. Then, the data distribution was verified using the Shapiro-Wilk test. Finally, to determine the significant differences between the means of the groups, the ANOVA tests were performed followed by the Bonferroni multiple comparison post-test (for data with parametric distribution) and the Kruskal–Wallis test followed by the Dunn multiple comparison post-test (for data with non-parametric distribution). Correlation analysis between antibody production and parasite burden was performed using the Spearman test. Statistical differences were considered significant when the p-value was less than or equal to 0.05. Dimensional reduction analyses (PCA and Heatmap) were performed using clustvis webtool [20]. PCA were calculated using Singular Value Decomposition Method (SVD). Both PCA and Heatmap include data of Frequency, Larvae Recovery, Number of Eosinophils, Neutrophils, Macrophages, Lymphocytes, total Leukocytes, IL-5, IL-13, IFNγ, TNFα, Hemoglobin, Total Protein, EPO, MPO, NAG, IgG Total, IgG1, IgG2a, IgG2b, IgG3, IgA, Lung resistance, Dynamic Compliance, Peribronchial Inflammation (Score), Perivascular Inflammation (Score), Parenchyma Inflammation (Score) and Haemorrhage (Score). In the Heatmap each number is one experimental animal and the colors represent the experimental groups.

## Results

### The impact of reinfections on parasite burden and antibody levels in *A*. *suum* infection

Previous studies carried out by our group have already examined animals exposed three times (multiple exposures) to *A*. *suum*, revealing a notable reduction in parasite burden [8]. However, to obtain a more comprehensive understanding of the impact of parasite reinfection, we initially sought to evaluate the role of different exposure frequencies in controlling parasite burden. Thus, mice were initially infected with *A*. *suum* eggs and subsequently the number of larvae recovered in the lungs and airways on the eighth-day post-challenge was compared between the single-infected group (SI) and the reinfected groups (RE 2x and RE 3x).

Our findings indicate no difference in the number of larvae recovered in the groups infected twice or thrice. Reinfected mice (RE 2x and RE 3x) exhibited a substantial reduction in total parasite burden (including tissue and bronchoalveolar lavage) of 95.6% and 99.2%, respectively, when compared to the single-infected (SI) group (Fig 1C–1E). Therefore, the results suggest effective control of the parasitic burden can be achieved with just two exposures, using the dose of 2,500 fully embryonated eggs.

When we checked whether there was a correlation between total specific IgG and SIgA depending on the parasite burden, we found a strong negative correlation between the levels of total IgG antibodies (Fig 1F) and SIgA (Fig 1G) and the parasite burden.

In the next step, we tried to observe the role of the different doses of eggs administered in the infection dynamic. Mice were infected twice with varying doses of *A*. *suum* eggs (RE 25, RE 250 and RE 2500) and, on the third time, were challenged with a dose of 2.500 eggs. Subsequently, the number of larvae recovered in the lungs and airways on the eighth-day post-challenge was compared between the single-infected group (SI) and the reinfected groups (RE 25, RE 250 and RE 2500) (Fig 1H–1J). Our results evidenced a significant dose-dependent reduction in mice from the reinfected groups of 48,4% (RE 25), 98,6% (RE 250) and 99,0% (RE 2500) compared to the single-infected group (SI) (Fig 1H–1J).Thus, as observed with the different exposure frequencies, the animals at different doses showed a decrease in parasite burden and an increase in total IgG (Fig 1K) and SIgA (Fig 1L) antibody levels.

**Fig 2:**
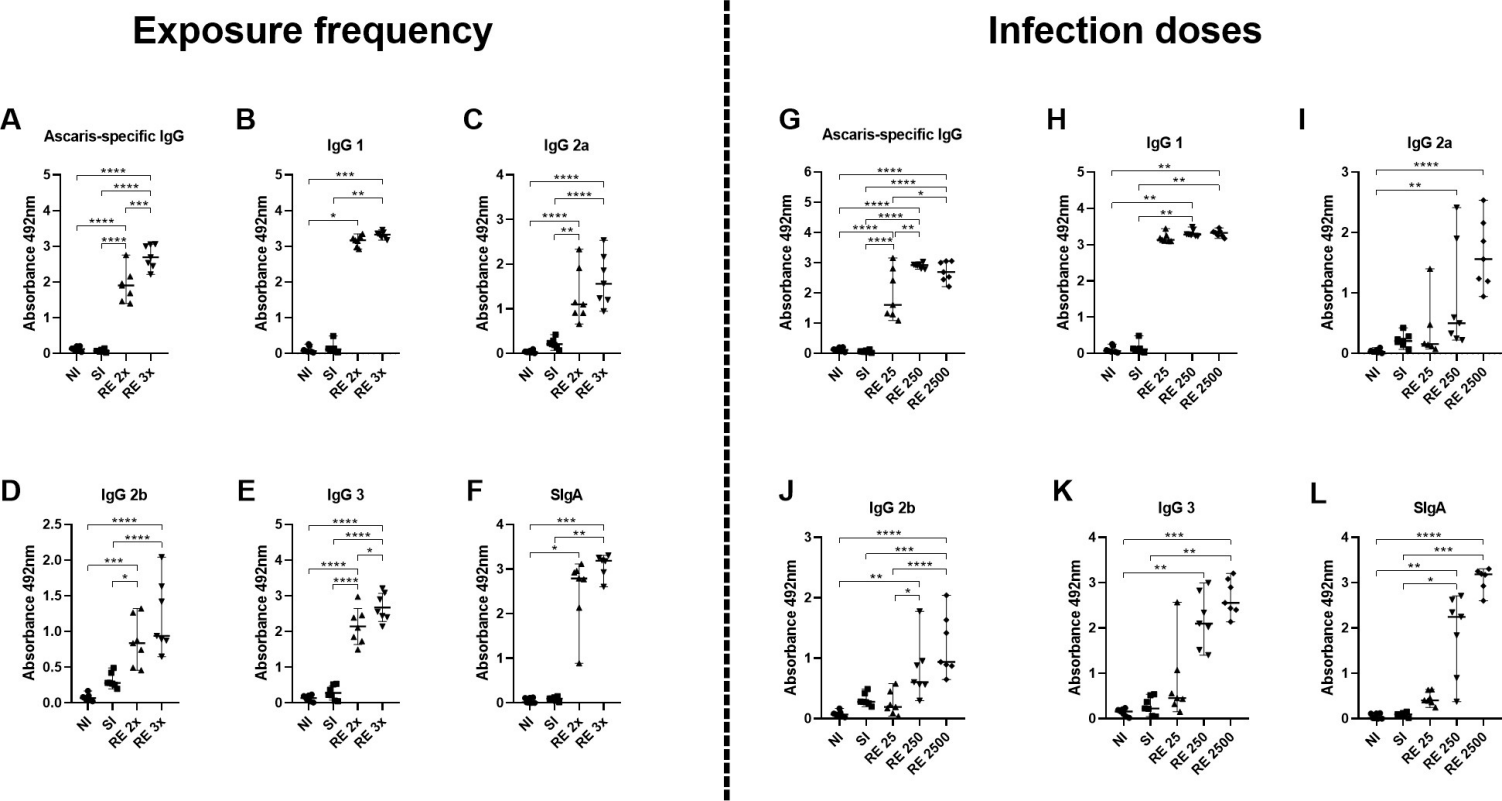
Serum levels of total IgG and subtypes and levels of SIgA in BAL according to exposure frequency and infection dose. (A and G) Total IgG; (B and H) IgG1; (C and I) IgG2a; (D and J) IgG2b; (E and K) IgG3; (F and L) SIgA. Significant differences between groups are indicated with (*). Where *p<0.05, **p<0.01, ***p<0.001, ****p<0.0001. Results are presented in graphs with a median and 95% CI.

To comprehend the correlation between various scenarios and the development of immunological memory in infected mice, we quantified not only the total specific IgG levels (Fig 2A and 2G) but also the IgG subclasses (IgG1, IgG2a, IgG2b, IgG3) in the animal serum (Fig 2B–2E and 2H–2K) and SIgA in BAL (Fig 2F and 2L). In general, a significant increase in the titers of antigen-specific IgG (Fig 2A), IgG1 (Fig 2B), IgG2a (Fig 2C), IgG2b (Fig 2D), IgG3 (Fig 2E), and IgA (Fig 2F) was observed in the RE 2x and RE 3x reinfected groups when compared to the negative control (NI) and control groups of single-infected animals (SI). The profile remains consistent when analyzing the impact of different infection doses. Reinfected animals, particularly RE 250 and RE 2500, showed a significant increase in antibody production compared to the control groups.

### The reinfection inflammatory profile indicates increased leukocyte recruitment and cytokine expression, as well as reduced hemorrhage in BAL

To investigate whether two exposures to the parasite and different doses administered to the host would increase leukocyte recruitment and a possible protective response, we characterized airway and tissue leukocyte flow.

**Fig 3:**
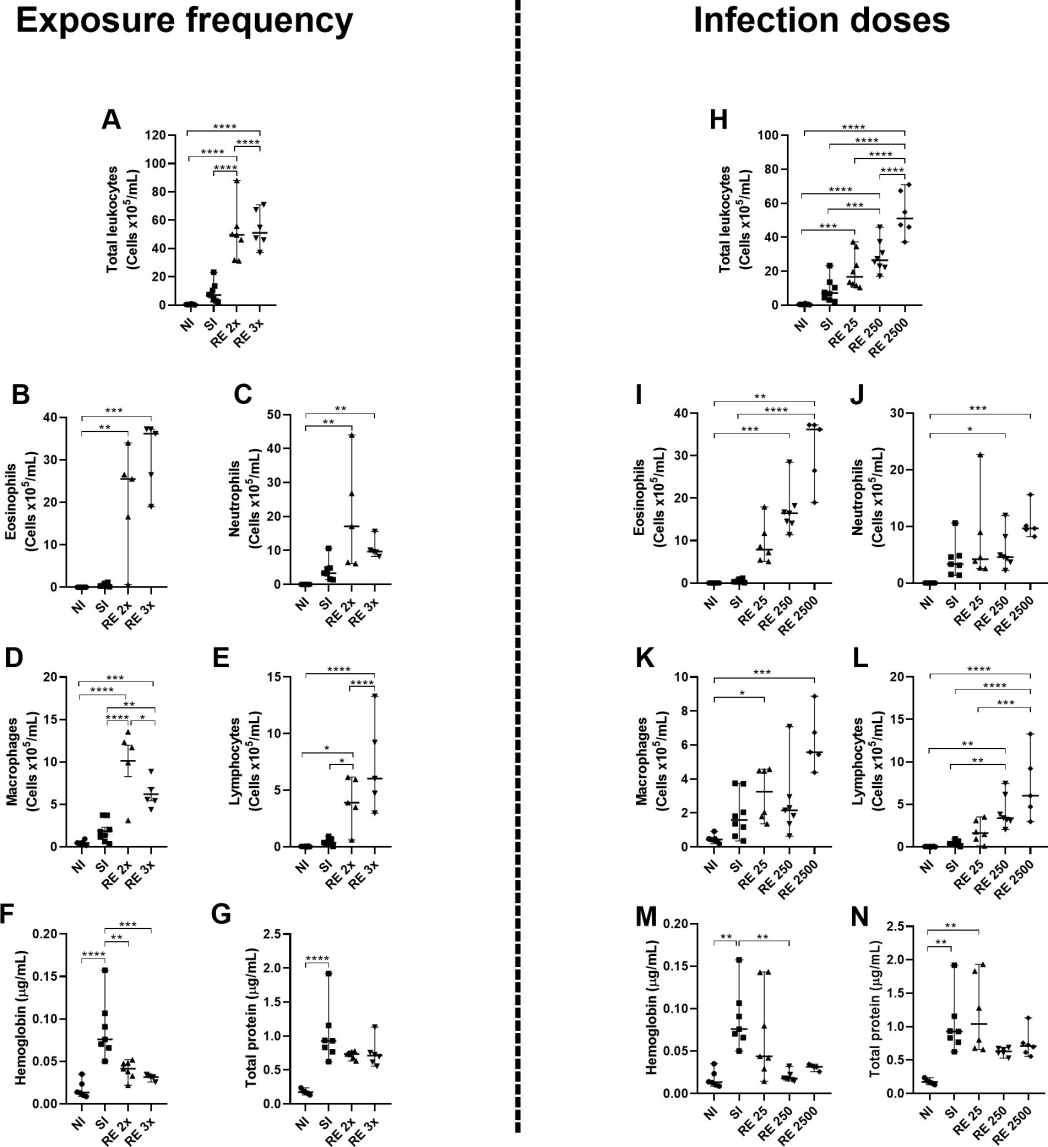
Characterization of inflammatory aspects and damage after different exposure frequencies and different infection doses. Leukocyte recruitment in BAL was quantified using a Neubauer chamber and cytospin preparations. (A and H) Total leukocytes; (B and I) eosinophils; (C and J) neutrophils; (D and K) macrophages; (E and L) lymphocytes; (F and M) hemoglobin; (G and N) total protein. Significant differences between groups are indicated with (*). Where *p<0.05, **p<0.01, ***p<0.001, ****p<0.0001. Results are presented in graphs with a median and 95% CI.

Considering the increased frequency of exposure, a significant increase in the total flow of leukocytes (Fig 3A) and cell subtypes (Fig 3B–3E) was observed in the reinfected animals (RE 2x and RE 3x) compared to the no-infected control (NI) and single-infected (SI) groups, with an increase in the infiltrate of eosinophils, macrophages and lymphocytes in these groups (Fig 3B, 3D and 3E).

Concerning the different doses of infection, there was a gradual increase in the total number of leukocytes, peaking in the RE2500 group (Fig 3H). This gradual increase was particularly reflected in the number of eosinophils (Fig 3I). For neutrophils and lymphocytes, only the RE250 and RE2500 groups showed statistically significant differences, with higher numbers compared to the NI group (Fig 3J and 3L). As for macrophages, only the RE25 and RE2500 groups displayed statistically significant differences when compared to the NI animals (Fig 3K). In addition to cellularity, the presence of hemoglobin in the animals’ airways was checked. In both, exposure frequency and infection dose analyses, the single-infected group (SI) exhibited a significant increase in the presence of hemorrhage compared to the groups of animals reinfected multiple times with RE 2x and RE 3x (Fig 3F) and the groups reinfected with different doses of RE 25, RE 250 and RE 2500 (Fig 3M). This suggests that the reinfected animals had less hemorrhage than the single infected group because fewer larvae migrated through the lungs, thus controlling the parasite burden. The single-infected group (SI) and RE 25 showed a high exudation of proteins. In contrast, in both analyses, the other reinfected groups tended to reduce protein presence (Fig 3G and 3N).

**Fig 4:**
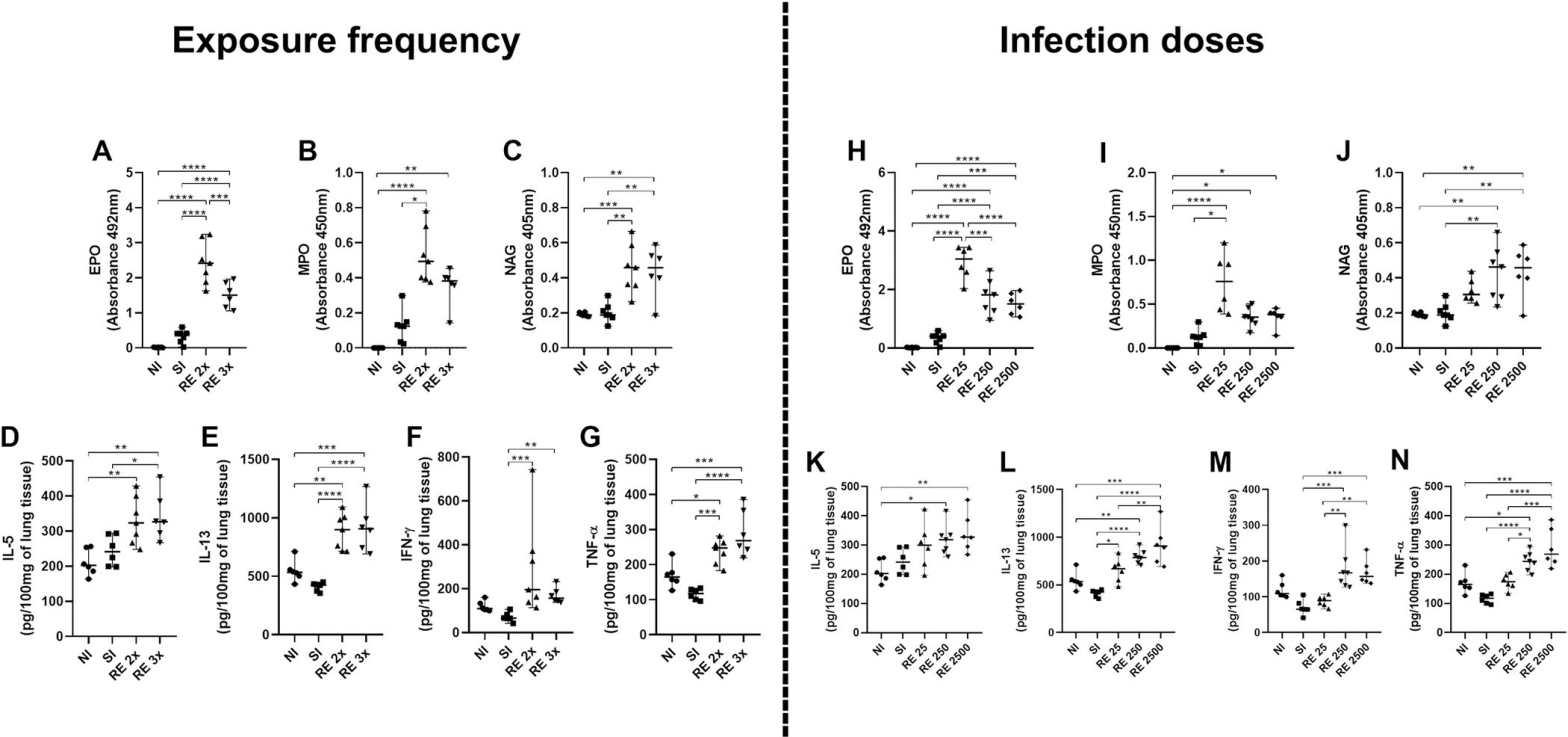
Levels of cellular activity and pulmonary cytokines after infection with *A*. *suum*. Cytokine levels were quantified by ELISA: (A and H) EPO; (B and I) MPO; (C and J) NAG; (D and K) IL-5; (E and L) IL-13; (F and M) IFN-γ; (G and N) TNF-α. Significant differences between the groups are represented by (*). Where *p<0.05, **p<0.01, ***p<0.001, ****p<0.000. The results are presented in the graphs with a median with 95% CI.

An indirect assessment of the activity of eosinophils, neutrophils and macrophages in the lung tissue showed an increase in EPO activity in the reinfected groups when compared to the no-infected (NI) and single-infected (SI) control groups, but with a more marked increase in the RE 2x group when compared to the RE 3x group (Fig 4A) and in the RE 25 group when compared to the RE 250 and RE 2500 groups (Fig 4H). There was also an increase in neutrophil activity in animals from the reinfected groups compared to the NI group. Among the reinfected groups, the RE 2x and RE 25 groups also showed a significant increase compared to the single-infected group (SI) (Fig 4B and 4I). Similarly, the reinfected groups had more significant NAG activity than the NI and SI groups (Fig 4C).

After confirming the increase in cellularity, we quantified the cytokines in the lung tissue. Our observations showed an increase in the production of cytokines with a mixed profile in the reinfected animals. Specifically, there was an increase in the levels of the cytokine IL-5 (Fig 4D) and IL-13 (Fig 4E) in relation to the number of exposures to infection, as well as an increase in these cytokines related to the increase in the dose of infection (Fig 4K and 4L). This increase is also seen in the dosages of the pro-inflammatory cytokines IFN-γ and TNF-α, especially in the groups reinfected with RE 2x, RE 3x (Fig 4F and 4G) and RE 250 and RE 2500 (Fig 4M and 4N).

### The reinfection to *A*. *suum* intensifies lung inflammation while simultaneously reducing damage in the lung, without significantly altering physiology among groups

**Fig 5:**
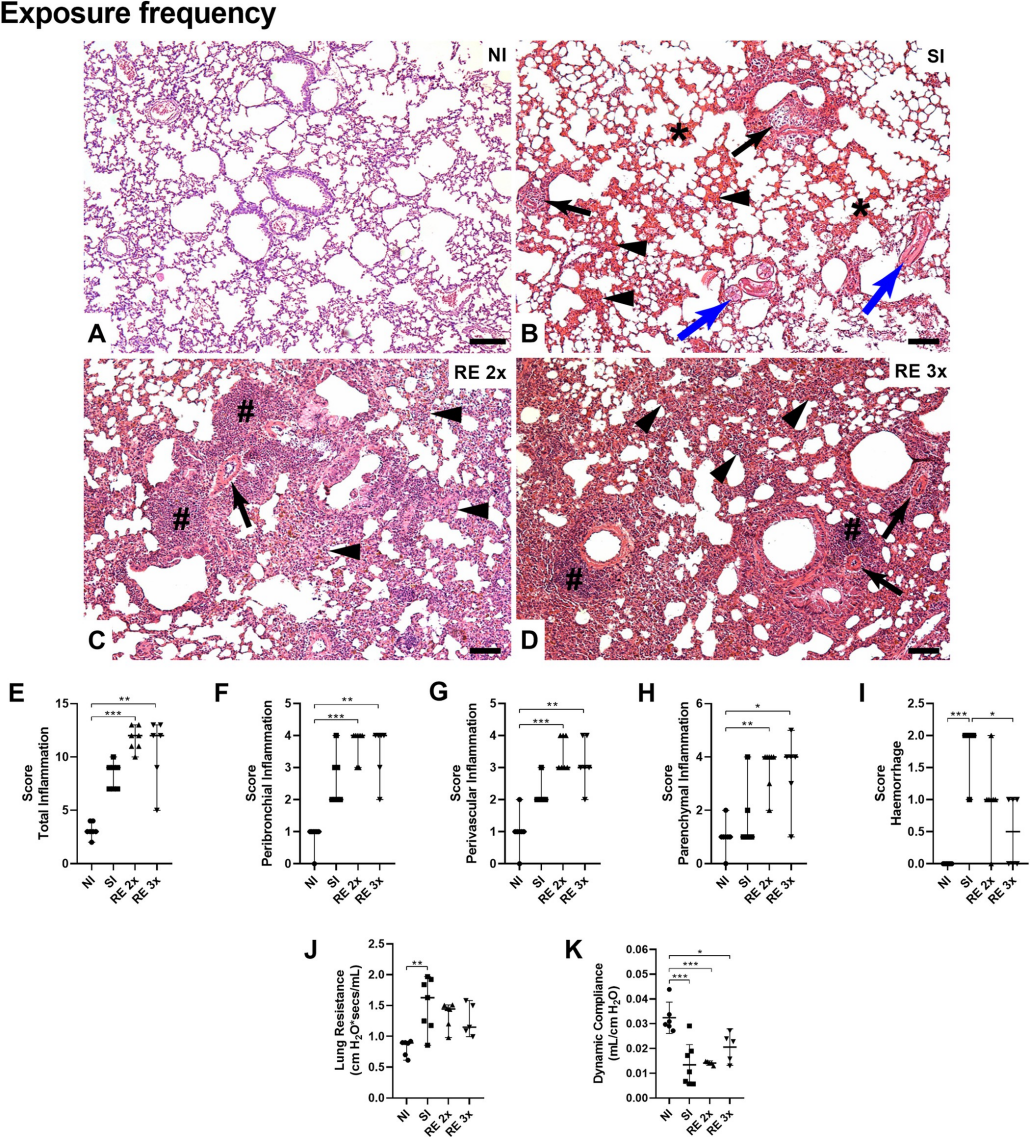
Histopathological inflammatory and physiological pulmonary changes in the lungs of mice (at 8dpi) with different frequencies exposures. (A) NI: Lung parenchyma with usual appearance; (B) SI: Lung parenchyma showing slight thickening of interalveolar septa (arrow heads), perivascular edema (arrow), L3 pulmonary larvae of *A*. *suum* (blue arrow), presence of exuberant hemorrhagic zones (*); (C) RE 2x: Lung parenchyma showing great thickening of interalveolar septa (arrow heads), perivascular edema (arrow), massive presence of inflammatory infiltrate around the lower airways and blood vessels (#); (D) RE 3x: Lung parenchyma showing great thickening of interalveolar septa (arrow heads), perivascular edema (arrow), massive presence of inflammatory infiltrate around the lower airways and blood vessels (#);. Bar = 100μm. Hematoxylin & Eosin staining. Semi-quantitative assessment of lung inflammation and presence of hemorrhage. (E) Total Inflammation Score; (F) Peribronchial Inflammation; (G) Perivascular Inflammation; (H) Parenchymal Inflammation; (I) Hemorrhage; (J) Pulmonary Resistance; (K) Dynamic Compliance. Significant differences between the groups are represented by (*). Where *p<0.05, **p<0.01, ***p<0.001, ****p<0.0001. The results are shown in the graphs with a median and 95% CI.

**Fig 6:**
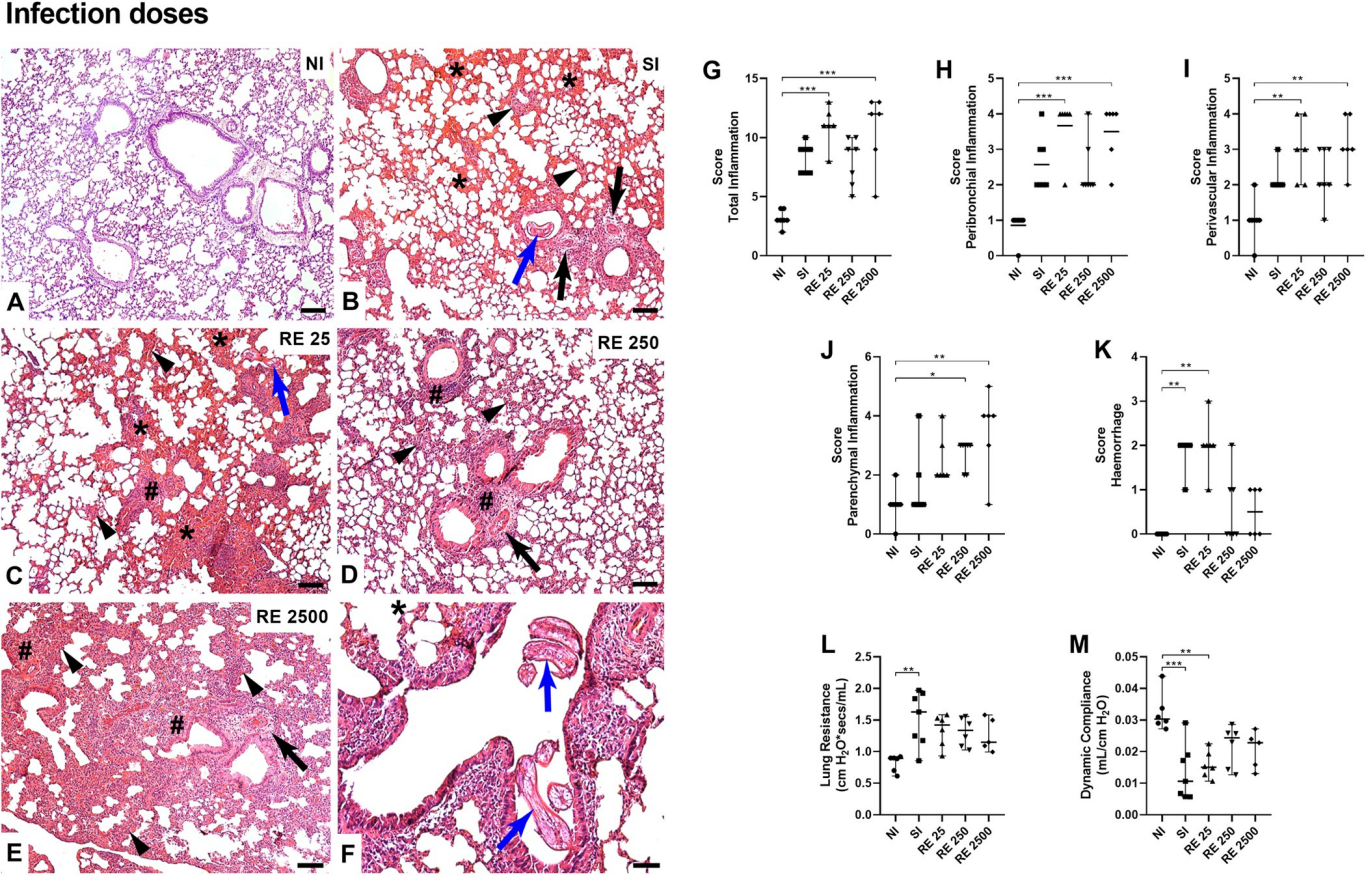
Histopathological inflammatory and physiological pulmonary changes in the lungs of mice (at 8dpi) with different infection doses. (A) NI: Lung parenchyma with usual appearance; (B) SI: Lung parenchyma showing slight thickening of interalveolar septa (arrow heads), perivascular edema (arrow), L3 pulmonary larvae of *A*. *suum* (blue arrow), presence of exuberant hemorrhagic zones (*); (C) RE 25: Lung parenchyma showing mild to moderate thickening of the interalveolar septa (arrowheads), presence of inflammatory infiltrate around the blood vessels (#), pulmonary L3 larva of *A*. *suum* (blue arrow), presence of exuberant hemorrhagic zones (*); (D) RE 250: Lung parenchyma showing slight thickening of the interalveolar septa (arrow heads), perivascular edema (arrow), presence of inflammatory infiltrate around the lower airways and blood vessels (#); (E) RE 2500: Lung parenchyma showing great thickening of interalveolar septa (arrow heads), perivascular edema (arrow), massive presence of inflammatory infiltrate around the lower airways and blood vessels (#); (F) Higher magnification image showing details of the pulmonary L3 larva of *Ascaris suum* (blue arrow) within the lumen of a bronchus of an animal from group RE 25. Bar = 100μm. Hematoxylin & Eosin staining. Semi-quantitative assessment of lung inflammation and presence of hemorrhage. (G) Total Inflammation Score; (H) Peribronchial Inflammation; (I) Perivascular Inflammation; (J) Parenchymal Inflammation; (K) Hemorrhage; (L) Pulmonary Resistance; (M) Dynamic Compliance. Significant differences between the groups are represented by (*). Where *p<0.05, **p<0.01, ***p<0.001, ****p<0.0001. The results are shown in the graphs with a median and 95% CI.

After observing increased cellularity in the lungs of the animals, we evaluated their histology. This allowed us to follow the pathological changes resulting from reinfection. An increase in the total inflammation score was observed in the airways, around the vessels, and in the parenchyma of the reinfected animals (Figs 5E–5I and 6G–6K); the presence of more intense hemorrhage in the SI and RE 25 groups, being significantly more remarkable when compared to the NI and RE 3x groups (Figs 5I and 6K).

Additionally, the histological panel revealed significant thickening of the interalveolar septa and an increased inflammatory infiltrate surrounding the airways and blood vessels, particularly in the RE 2x and RE 3x reinfected groups (Figs 5A–5D and 6A–6E). Notably, among the reinfected groups, the RE 250 group compared to the RE 25 and RE 2500 groups showed a more discreet inflammatory infiltrate and slight thickening of the septa. All these results corroborate those observed in the EPO, MPO, and NAG analyses and the identification of cells in BAL.

After verifying the reduced parasite burden and increased pulmonary inflammation in the reinfected groups, we performed spirometry on the animals to assess pulmonary physiological parameters (at 8dpi). It was possible to see that there were no significant differences in compliance and resistance parameters between the infected groups; however, there was a substantial increase in resistance in the SI groups compared to the NI group (Fig 5J) and a significant decrease in compliance in the other groups compared to the NI group (Fig 5K). As for the impact of reinfections with different doses on lung function, it was also possible to observe greater resistance in the SI group compared to the NI group (Fig 6L) and a decrease in compliance in the SI and RE 25 groups when compared only to the NI group (Fig 6M).

### Principal component analysis (PCA) supports the differences between the frequency of exposure and the dose of infection

**Fig 7:**
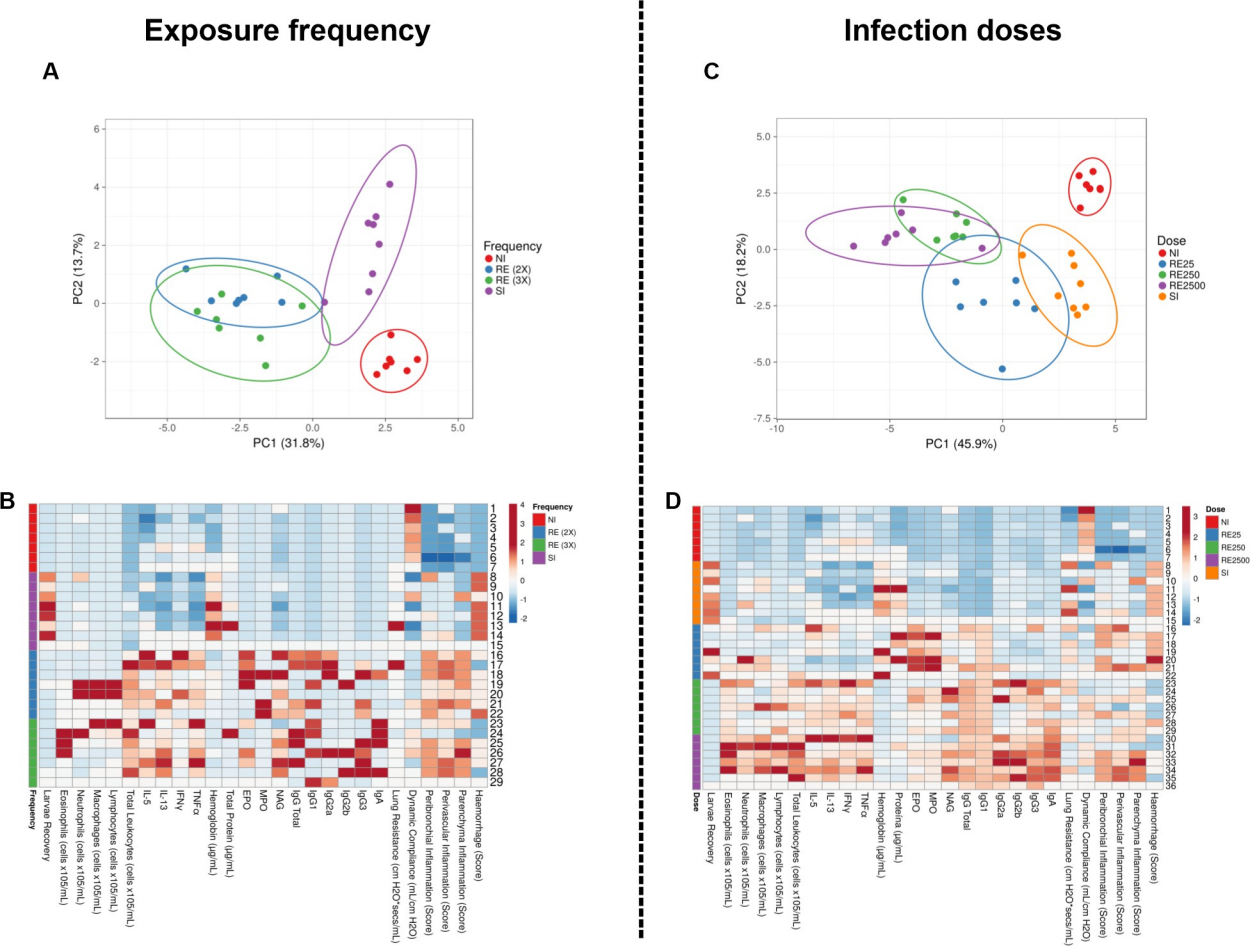
Principal Component Analysis (PCA) and Heatmap of the different analyses performed. Principal Component Analysis (PCA) confirms the changes and similarities between the groups exposed to different infection conditions and Heatmap shows the analysis of the inflammatory profile and lung function in infected mice. Both were carried out with the values for all the variables analyzed in this study, where (A) PCA corresponds to the exposure frequency NI (Red), RE 2x (Blue), RE 3x (Green), SI (Purple); (B) Heatmap corresponds to the exposure frequency NI (Red), RE 2x (Blue), RE 3x (Green), SI (purple); (C) PCA corresponds to the infection dose NI (Red), RE 25 (Blue), RE 250 (Green), RE 2500 (Purple), SI (Orange); (D) Heatmap corresponds to the infection dose NI (Red), RE 25 (Blue), RE 250 (Green), RE 2500 (Purple), SI (Orange).

To evaluate the heterogeneity between the groups, we conducted a dimensional reduction technique through Principal Component Analysis (PCA) using all the variables analyzed in the study (Fig 7A and 7C). Upon examining the PCA segregation for principal component 1 (PC1) vs. principal component 2 (PC2), it was possible to observe a clear and well-defined separation of NI and SI group and a proximity between the reinfected groups. Thus, our PCA confirms that the infection dynamics of *A*. *suum* vary according to the frequency of exposure and the dose administered. In addition, the PCA in Fig 7C reveal a systematic change in the clusters based on the infection dose. First, there is a clear separation between the samples with no-infected (NI) and those that are infected. Secondly, the data confirm the separation between single-infected (SI) samples and reinfected (RE) samples. Among the samples with higher infection doses (RE 250 and RE 2500), there is a reduced separation, suggesting a similarity in their response to the higher infection doses compared to RE 25 and SI samples.

Beyond the PCA, all variables were displayed on a heatmap (Fig 7B and 7D) to synthesize and improve the visualization of our results. We present the results obtained in relation to the frequency of exposure and the dose of infection. The image allows us to clearly represent in a graphic and concise way the role that these scenarios play in reducing parasite burden, cytokine expression, lung physiology and host damage. The first heatmap analysis clearly demonstrates a significant reduction in larvae recovery, IFNγ, TNF, and hemorrhage in the reinfected (RE) groups compared to single-infected (SI) counterparts. Shades of blue dominate the RE columns, indicating lower values in these parameters, signifying a potential immunomodulatory effect upon reinfection. Conversely, the heatmap reveals an upregulation in total leukocytes, Eosinophil Peroxidase (EPO), Myeloperoxidase (MPO), N-Acetyl-beta-D-Glucosaminidase (NAG), and humoral responses (total IgA, IgG, IgG subclasses) in the RE groups. Shades of red dominate the RE columns, indicating heightened immune activity and robust humoral responses in the face of reinfection.

As the reinfection dose increases, there is a consistent rise in total leukocytes, reflecting an intensified host immune response. Concurrently, the cytokine expression profile demonstrates a gradual elevation in IL5, IL13, IFNγ, and TNF, indicating an increasingly complex immunomodulatory environment according to the infection dose. NAG activity and humoral responses, measured by total IgG and IgA, also exhibit an upward trend with escalating reinfection doses. Interestingly, the reinfection group with the lower dose (RE 25) stands out as the peak of hemorrhage, protein content, EPO, and MPO activity. This observation indicates a maximal pathological response at the RE 25 dose, characterized by heightened vascular permeability, protein leakage, and eosinophil and neutrophil activity. This inverse relationship between infection dose and hemorrhage suggests a potential adaptive host response or a shift in the parasite’s impact on vascular integrity at higher infection doses. The observed patterns in the heatmap underscore the intricate balance between host immunity and pathological responses during *A*. *suum* reinfection. The immunological and pathological parameters demonstrate a nuanced interplay, with certain responses peaking at intermediate doses, highlighting the complexity of the host-parasite interaction in this experimental model. The heatmap and PCA analysis were valuable tools for highlighting trends and patterns, contributing significantly to the interpretation of the data and the identification of relevant correlations in the different exposure scenarios and infection doses studied.

## Discussion

In this study, we delved into the immunological and pathophysiological aspects associated with the exposure frequency and dose infection of *A*. *suum* eggs in a murine model. This study demonstrates that the intensity of the damage caused to the host and the protective mechanisms related to reducing the parasite burden are influenced by the dose administered and the frequency of exposure to the parasite.

To date, works regarding the dynamics of *Ascaris* spp. infections are limited. Most murine studies involve a single exposure to a high standard dose of 2,500 eggs [9,10,21,22]. Although these studies are relevant in understanding the immune response, they do not reflect natural conditions where multiple low dose occur [12,23]. Moreover, experimental evaluations on multiple low dose nematodes infections are scarce [24–26], particularly for *Ascaris* spp. [27]. Only one study describes a reinfection scenario in an experimental model, but the gaps remain, since the same dose of eggs (2,500) was used [8].

Our results expand the understanding of *A*. *suum* infections, shedding light on the heterogeneity of helminth establishment in the field where the majority of individuals in endemic areas have continuous exposure to the parasite and often exhibit a low parasite burden throughout their lives [26,28–31]. When evaluating the frequency of exposures, our results revealed that two exposures were enough to cause a significant reduction in the parasite burden, corroborating previous findings on multiple exposures [8]. Evidence that a single previous exposure to helminths can induce protection has been observed in other experimental models [30,32–35], including *A*. *suum* in non-murine models [36–38]. Explanations related to parasite burden control in reinfection scenarios are controversial. Some authors attribute this relationship to the humoral response due to an increased in antibody levels [39,40], while others relate the protection to a reflection of the intensity of cellularity induced by the infection [8,41–45]. We suggest an association between the decrease in parasite burden and the intensity of cellularity, and we observed a significant increase in leukocytes in the airways of reinfected animals depending on the frequency and dose of infection, similar to that observed in previous studies with multiple infections [8,45].

The effector role of eosinophils in controlling parasite burden during helminth infection has been widely discussed [45–50]. Furthermore, Nogueira et al. (2021) [45] observed that the absence of these cells impaired parasite load control in *A*. *suum* infections. In this study, we observed a consistent increase in eosinophils regardless of the frequency of exposure, but with varying intensity depending on the dose administered. We also noted an increase in neutrophils and cytotoxic granules (myeloperoxidase) in the airways of reinfected animals. The role of neutrophils as important effector cells in limiting parasite survival and dissemination in murine models of helminth infection has been described in the literature, serving as the host’s first line of defense [51–54].

The involvement of macrophages in *A*. *suum* infections has been previously documented [10,11,22,50] and recent studies suggest the importance of these cells in the continuous activation of the immune system through frequent phagocytosis of *A*. *suum* antigens [11]. Indeed, the higher number of these cells observed in animals infected twice compared to those infected three times was unexpected and further characterization of this cell type is needed to better understand this phenomenon and its implications for the immune response in repeated infections. The lymphocytosis observed in this study can be attributed to the adaptive immune response, especially the expansion of B cells and the production of antibodies, as well as the establishment of immunological memory. However, a more detailed characterization of these lymphocytes is needed, including the identification of specific T and B cell subtypes and their cytokine profiles, to better elucidate the dynamics of the immune response.

The increased inflammation and cellularity observed in reinfected animals are in accordance with findings that demonstrate that there is an increase in inflammatory markers in children immune to infection compared to infected children [40]. Furthermore, it is possible that this overall inflammation observed in reinfection may have long-term effects. Oliveira and colleagues demonstrated in a single infection experimental model of *A*. *suum* that cellularity increases at early time points, such as 8 dpi; however, fibrosis only emerges later, at 21 and 28 dpi [19]. Additionally, the pulmonary physiological changes were persistent throughout the entire period analyzed [19]. Additionally, the pulmonary physiological changes were persistent throughout the entire period analyzed [19]. Studies considering only one exposure [18,19] showed a decrease in lung compliance and an increase in resistance. Interestingly, our results indicate that although there was no significant difference between the reinfected groups, there was a tendency for the elastic capacity of the lungs to recover in the RE 250, RE 3x, and RE 2500 animals. This could be attributed to reduced larval migration in the tissue and less hemorrhage observed in those animals. It is important to highlight that different infectious doses may impact the dynamics of parasite migration to the lungs, potentially affecting larval recovery and the damage observed at later time points. Future studies evaluating how reinfection and different infectious doses may impact the scenario of chronic damage are necessary.

Results described by Gazzinelli-Guimarães et al. (2018) [18], show that there is an increase in total antibody levels in animals exposed to *A*. *suum* antigens. This led us to assume that parasite burden control might be associated with an increase in antibody levels. Thus, our data showed that the higher the frequency of exposure, the higher the antibody levels. In terms of doses, we observed that two exposures at lower doses (25 eggs) did not reduce parasite burden as much as doses 250 and 2,500 but significantly increased antibody levels compared to a single exposure at 2,500 eggs. In this case, we believe that at least three or more exposures would be required to achieve results similar to the 250 and 2,500 egg doses, including reducing tissue parasite burden. The data suggests that the generation of immunological memory established a relationship with reduced larval recovery rates.

Besides the involvement of antibodies, several studies have established a relationship between the control of parasite burden in various helminthic infections and the production of cytokines [55–58]. In our study, we demonstrated the involvement of type 2 (IL-5 and IL-13) and type 1 (IFN-γ and TNF) cytokines after two exposures to the parasite, similar to animals exposed three times to a single dose of 2,500 eggs [8].

It has been described that the presence of helminth larvae in the lung triggers rapid activation and recruitment of eosinophils through the release of chemoattractant eotaxins, and the production of IL-5 [59–62]. During larval ascariasis, both in a simple infection model [9], and in the present study, an increase in the cytokine IL-5 was observed in lung tissue, which is probably related to the higher number and activity of eosinophils in reinfected mice, since IL-5 increases the differentiation, maturation and survival of eosinophils derived from bone marrow precursors [63,64]. Curiously, IL-5 is also described as a potent inducer of B cell differentiation, antibody secretion, and isotype switching [65]. Thus, a protective Th2 response induced by immunization may be secondary to a specific IgG humoral response induced by IL-5 [18]. Our data corroborates with that observed by Glover et al., (2019) [26], which analyzed repeated infections with low doses ("trickle infection") of *Trichuris muris* eggs. They showed that was a slow acquisition of immunity and development of resistance over time, significantly reducing the number of worms, but without total elimination of the infection due to the persistence of a low parasite burden. Our data, together, suggest that the number of low-dose infection events is key to generating resistance and not simply the slow development of protective immunity.

## Conclusion

**Fig 8:**
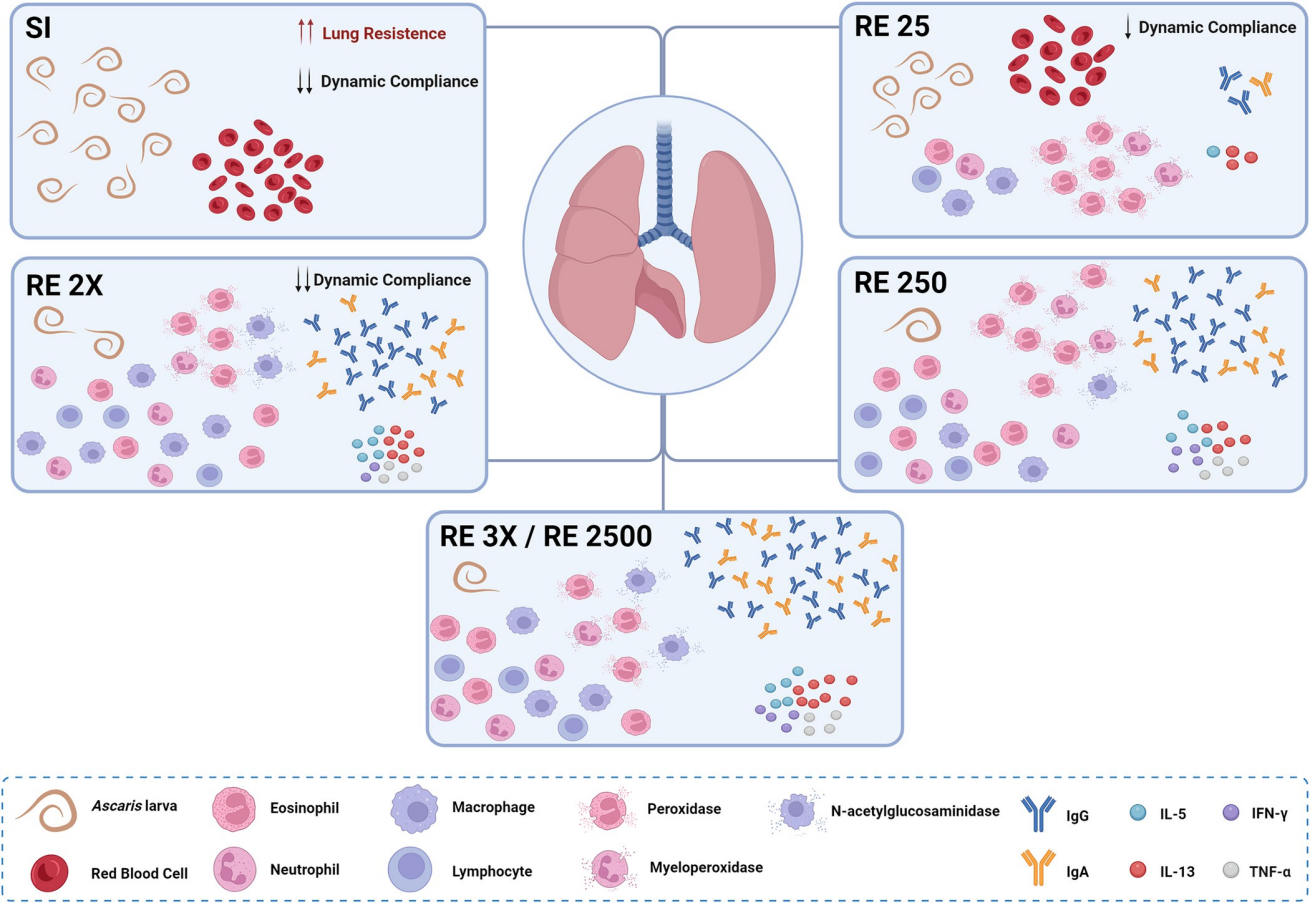
Schematic representation of the *Ascaris suum* infections scenario with different exposure frequencies and infection doses on the eighth day post-infection. Figure created in BioRender (BioRender.com/i72s52).

Based on the present study, it is evident that the assessed parameters appear to exhibit a dose-response effect. It is crucial to highlight that when contrasting the two conditions under investigation in the study, namely exposure frequency and infection doses, the RE 2x and RE 250 groups yielded similar results in the majority of the analyses (Fig 8). The primary distinction between the two groups lies in the greater histopathological impairment, such as greater septal thickening, and physiological impairment, such as reduced compliance, exhibited by the RE 2x group. Furthermore, recognizing the limitations of the murine model in ascariasis studies, the use of two doses of 250 eggs seems to be closer to natural conditions, resulting in a patent infection with reduced parasite burden (similar to the groups reinfected with high doses—RE 3x and RE 2500) and consequent less physiological damage. This result provides us with a promising perspective and indicates that exposure to lower doses might be sufficient to control the migration of the larvae in the lungs without causing significant damage. Additionally, we highlight that other studies exploring the role of these infectious doses and effects after a peak of larval migration should be conducted, including humoral response, parasite burden control after more exposures and damage repair in tissue.

## Supporting information

S1 Fig Histopathological scoring system for lungs. (TIFF)

S1 Table Analyses and respective comparative P-values. (PDF)

## References

[1] Holland C, Sepidarkish M, Deslyper G, Abdollahi A, Valizadeh S, Mollalo A, et al. Global prevalence of Ascaris infection in humans (2010–2021): a systematic review and meta-analysis. Infect Dis Poverty. 2022;11: 113. doi: 10.1186/s40249-022-01038-z 36401308 PMC9673379

[2] Ascariasis—Level 4 cause | Institute for Health Metrics and Evaluation. [cited 25 Jan 2024]. Available: https://www.healthdata.org/results/gbd_summaries/2019/ascariasis-level-4-cause

[3] Jia T-W, Melville S, Utzinger J, King CH, Zhou X-N. Soil-transmitted helminth reinfection after drug treatment: a systematic review and meta-analysis. PLoS Negl Trop Dis. 2012;6: e1621. doi: 10.1371/journal.pntd.0001621 22590656 PMC3348161

[4] Avery RH, Wall LA, Verhoeve VI, Gipson KS, Malone JB. Molecular Confirmation of *Ascaris suum*: Further Investigation into the Zoonotic Origin of Infection in an 8-Year-Old Boy with Loeffler Syndrome. Vector Borne Zoonotic Dis. 2018;18: 638–640. doi: 10.1089/vbz.2018.2306 30085905

[5] Sadaow L, Sanpool O, Phosuk I, Rodpai R, Thanchomnang T, Wijit A, et al. Molecular identification of *Ascaris lumbricoides* and *Ascaris suum* recovered from humans and pigs in Thailand, Lao PDR, and Myanmar. Parasitol Res. 2018;117: 2427–2436. doi: 10.1007/s00436-018-5931-6 29860571

[6] Silva TED, Barbosa FS, Magalhães LMD, Gazzinelli-Guimarães PH, Dos Santos AC, Nogueira DS, et al. Unraveling *Ascaris suum* experimental infection in humans. Microbes and Infection. 2021;23: 104836. doi: 10.1016/j.micinf.2021.104836 34020024

[7] Monteiro KJL, Calegar DA, Santos JP, Bacelar PAA, Coronato-Nunes B, Reis ERC, et al. Genetic diversity of *Ascaris* spp. infecting humans and pigs in distinct Brazilian regions, as revealed by mitochondrial DNA. PLoS One. 2019;14: e0218867. doi: 10.1371/journal.pone.0218867 31233550 PMC6590885

[8] Nogueira DS, Gazzinelli-Guimarães PH, Barbosa FS, Resende NM, Silva CC, de Oliveira LM, et al. Multiple Exposures to *Ascaris suum* Induce Tissue Injury and Mixed Th2/Th17 Immune Response in Mice. PLoS Negl Trop Dis. 2016;10: e0004382. doi: 10.1371/journal.pntd.0004382 26814713 PMC4729520

[9] Gazzinelli-Guimarães PH, Gazzinelli-Guimarães AC, Silva FN, Mati VLT, Dhom-Lemos L de C, Barbosa FS, et al. Parasitological and immunological aspects of early *Ascaris* spp. infection in mice. Int J Parasitol. 2013;43: 697–706. doi: 10.1016/j.ijpara.2013.02.009 23665127

[10] Wu Y, Li E, Knight M, Adeniyi-Ipadeola G, Song L-Z, Burns AR, et al. Transient *Ascaris suum* larval migration induces intractable chronic pulmonary disease and anemia in mice. PLoS Negl Trop Dis. 2021;15: e0010050. doi: 10.1371/journal.pntd.0010050 34914687 PMC8717995

[11] Oliveira FMS, Kraemer L, Vieira-Santos F, Leal-Silva T, Gazzinelli-Guimarães AC, Lopes CA, et al. The long-lasting *Ascaris suum* antigens in the lungs shapes the tissue adaptation modifying the pulmonary architecture and immune response after infection in mice. Microb Pathog. 2024;186: 106483. doi: 10.1016/j.micpath.2023.106483 38092133

[12] Crombie JA, Anderson RM. Population dynamics of *Schistosoma mansoni* in mice repeatedly exposed to infection. Nature. 1985;315: 491–493. doi: 10.1038/315491a0 4000276

[13] Boes J, Eriksen L, Nansen P. Embryonation and infectivity of *Ascaris suum* eggs isolated from worms expelled by pigs treated with albendazole, pyrantel pamoate, ivermectin or piperazine dihydrochloride. Vet Parasitol. 1998;75: 181–190. doi: 10.1016/s0304-4017(97)00197-0 9637219

[14] Lewis R, Behnke JM, Stafford P, Holland CV. The development of a mouse model to explore resistance and susceptibility to early *Ascaris suum* infection. Parasitology. 2006;132: 289–300. doi: 10.1017/S0031182005008978 16209722

[15] Barcelos LS, Talvani A, Teixeira AS, Vieira LQ, Cassali GD, Andrade SP, et al. Impaired inflammatory angiogenesis, but not leukocyte influx, in mice lacking TNFR1. J Leukoc Biol. 2005;78: 352–358. doi: 10.1189/jlb.1104682 15894588

[16] Russo RC, Garcia CC, Barcelos LS, Rachid MA, Guabiraba R, Roffê E, et al. Phosphoinositide 3-kinase γ plays a critical role in bleomycin-induced pulmonary inflammation and fibrosis in mice. J Leukoc Biol. 2011;89: 269–282. doi: 10.1189/jlb.0610346 21048214

[17] Guabiraba R, Russo RC, Coelho AM, Ferreira MAND, Lopes GAO, Gomes AKC, et al. Blockade of cannabinoid receptors reduces inflammation, leukocyte accumulation and neovascularization in a model of sponge-induced inflammatory angiogenesis. Inflamm Res. 2013;62: 811–821. doi: 10.1007/s00011-013-0638-8 23722450

[18] Gazzinelli-Guimarães AC, Gazzinelli-Guimarães PH, Nogueira DS, Oliveira FMS, Barbosa FS, Amorim CCO, et al. IgG Induced by Vaccination With *Ascaris suum* Extracts Is Protective Against Infection. Front Immunol. 2018;9: 2535. doi: 10.3389/fimmu.2018.02535 30473693 PMC6238660

[19] Oliveira FMS, da Paixão Matias PH, Kraemer L, Gazzinelli-Guimarães AC, Santos FV, Amorim CCO, et al. Comorbidity associated to *Ascaris suum* infection during pulmonary fibrosis exacerbates chronic lung and liver inflammation and dysfunction but not affect the parasite cycle in mice. PLoS Negl Trop Dis. 2019;13: e0007896. doi: 10.1371/journal.pntd.0007896 31765381 PMC6901262

[20] Metsalu T, Vilo J. ClustVis: a web tool for visualizing clustering of multivariate data using Principal Component Analysis and heatmap. Nucleic Acids Research. 2015;43: W566–W570. doi: 10.1093/nar/gkv468 25969447 PMC4489295

[21] Weatherhead JE, Porter P, Coffey A, Haydel D, Versteeg L, Zhan B, et al. *Ascaris* Larval Infection and Lung Invasion Directly Induce Severe Allergic Airway Disease in Mice. Infect Immun. 2018;86: e00533–18. doi: 10.1128/IAI.00533-18 30249744 PMC6246907

[22] Oliveira FMS, Kraemer L, Cavalcanti da Silva C, Nogueira DS, Gazzinelli-Guimarães AC, Gazzinelli-Guimarães PH, et al. Nitric oxide contributes to liver inflammation and parasitic burden control in *Ascaris suum* infection. Exp Parasitol. 2022;238: 108267. doi: 10.1016/j.exppara.2022.108267 35550886

[23] Srisawangwong T, Sithithaworn P, Sukkasaem P, Jintakanon D, Tesana S, Sithithaworn J, et al. Concomitant and protective immunity in mice exposed to repeated infections with *Echinostoma malayanum*. Exp Parasitol. 2011;127: 740–744. doi: 10.1016/j.exppara.2011.01.010 21272582

[24] Ovington KS. Trickle infections of *Nippostrongylus brasiliensis* in rats. Z Parasitenkd. 1986;72: 851–853. doi: 10.1007/BF00925109 3799017

[25] Brailsford TJ, Behnke JM. The dynamics of trickle infections with *Ancylostoma ceylanicum* in inbred hamsters. Parasitology. 1992;105 (Pt 2): 247–253. doi: 10.1017/s0031182000074175 1454423

[26] Glover M, Colombo SAP, Thornton DJ, Grencis RK. Trickle infection and immunity to *Trichuris muris*. PLoS Pathog. 2019;15: e1007926. doi: 10.1371/journal.ppat.1007926 31730667 PMC6881069

[27] Lewis R, Behnke JM, Stafford P, Holland CV. Dose-dependent impact of larval *Ascaris suum* on host body weight in the mouse model. J Helminthol. 2009;83: 1–5. doi: 10.1017/S0022149X08912402 19021921

[28] Holland CV, Asaolu SO, Crompton DW, Stoddart RC, Macdonald R, Torimiro SE. The epidemiology of *Ascaris lumbricoides* and other soil-transmitted helminths in primary school children from Ile-Ife, Nigeria. Parasitology. 1989;99 Pt 2: 275–285. doi: 10.1017/s003118200005873x 2594419

[29] Barbosa CS, Favre TC, Wanderley TN, Callou AC, Pieri OS. Assessment of schistosomiasis, through school surveys, in the Forest Zone of Pernambuco, Brazil. Mem Inst Oswaldo Cruz. 2006;101 Suppl 1: 55–62. doi: 10.1590/s0074-02762006000900009 17308748

[30] Schilter HC, Pereira ATM, Eschenazi PD, Fernandes A, Shim D, Sousa ALS, et al. Regulation of immune responses to *Strongyloides venezuelensis* challenge after primary infection with different larvae doses. Parasite Immunol. 2010;32: 184–192. doi: 10.1111/j.1365-3024.2009.01176.x 20398181

[31] Colombo SAP, Grencis RK. Immunity to Soil-Transmitted Helminths: Evidence From the Field and Laboratory Models. Front Immunol. 2020;11: 1286. doi: 10.3389/fimmu.2020.01286 32655568 PMC7324686

[32] Dawkins HJ, Grove DI. Immunisation of mice against *Strongyloides ratti*. Z Parasitenkd. 1982;66: 327–333. doi: 10.1007/BF00925349 7080613

[33] Yu JR, Hong ST, Chai JY, Lee SH. The effect of reinfection with *Neodiplostomum seoulensis* on the histopathology and activities of brush border membrane bound enzymes in the rat small intestine. Korean J Parasitol. 1995;33: 37–43. doi: 10.3347/kjp.1995.33.1.37 7735784

[34] Schopf LR, Hoffmann KF, Cheever AW, Urban JF, Wynn TA. IL-10 is critical for host resistance and survival during gastrointestinal helminth infection. J Immunol. 2002;168: 2383–2392. doi: 10.4049/jimmunol.168.5.2383 11859129

[35] Sohn W-M, Zhang H, Choi M-H, Hong S-T. Susceptibility of experimental animals to reinfection with *Clonorchis sinensis*. Korean J Parasitol. 2006;44: 163–166. doi: 10.3347/kjp.2006.44.2.163 16809966 PMC2532638

[36] McCraw BM. The development of *Ascaris suum* in calves. Can J Comp Med. 1975;39: 354–357. 1139416 PMC1277471

[37] Eriksen L, Nansen P, Roepstorff A, Lind P, Nilsson O. Response to repeated inoculations with *Ascaris suum* eggs in pigs during the fattening period. I. Studies on worm population kinetics. Parasitol Res. 1992;78: 241–246. doi: 10.1007/BF00931733 1534170

[38] Nejsum P, Thamsborg SM, Petersen HH, Kringel H, Fredholm M, Roepstorff A. Population dynamics of *Ascaris suum* in trickle-infected pigs. J Parasitol. 2009;95: 1048–1053. doi: 10.1645/GE-1987.1 19673589

[39] Hagel I, Lynch NR, Di Prisco MC, Rojas E, Pérez M, Alvarez N. *Ascaris* reinfection of slum children: relation with the IgE response. Clin Exp Immunol. 1993;94: 80–83. doi: 10.1111/j.1365-2249.1993.tb05981.x 8403522 PMC1534372

[40] McSharry C, Xia Y, Holland CV, Kennedy MW. Natural immunity to *Ascaris lumbricoides* associated with immunoglobulin E antibody to ABA-1 allergen and inflammation indicators in children. Infect Immun. 1999;67: 484–489. doi: 10.1128/IAI.67.2.484–489.1999 9916049 PMC96345

[41] Haswell-Elkins MR, Kennedy MW, Maizels RM, Elkins DB, Anderson RM. The antibody recognition profiles of humans naturally infected with *Ascaris lumbricoides*. Parasite Immunol. 1989;11: 615–627. doi: 10.1111/j.1365-3024.1989.tb00925.x 2616191

[42] Haswell-Elkins MR, Leonard H, Kennedy MW, Elkins DB, Maizels RM. Immunoepidemiology of *Ascaris lumbricoides*: relationships between antibody specificities, exposure and infection in a human community. Parasitology. 1992;104 Pt 1: 153–159. doi: 10.1017/s0031182000060893 1614731

[43] Palmer DR, Hall A, Haque R, Anwar KS. Antibody isotype responses to antigens of *Ascaris lumbricoides* in a case-control study of persistently heavily infected Bangladeshi children. Parasitology. 1995;111 (Pt 3): 385–393. doi: 10.1017/s0031182000081944 7567106

[44] King E-M, Kim HT, Dang NT, Michael E, Drake L, Needham C, et al. Immuno-epidemiology of *Ascaris lumbricoides* infection in a high transmission community: antibody responses and their impact on current and future infection intensity. Parasite Immunol. 2005;27: 89–96. doi: 10.1111/j.1365-3024.2005.00753.x 15882235

[45] Nogueira DS, de Oliveira LM, Amorim CCO, Gazzinelli-Guimarães AC, Barbosa FS, Oliveira FMS, et al. Eosinophils mediate SIgA production triggered by TLR2 and TLR4 to control *Ascaris suum* infection in mice. PLoS Pathog. 2021;17: e1010067. doi: 10.1371/journal.ppat.1010067 34784389 PMC8631680

[46] Padigel UM, Hess JA, Lee JJ, Lok JB, Nolan TJ, Schad GA, et al. Eosinophils act as antigen-presenting cells to induce immunity to *Strongyloides stercoralis* in mice. J Infect Dis. 2007;196: 1844–1851. doi: 10.1086/522968 18190266 PMC3154724

[47] Cadman ET, Lawrence RA. Granulocytes: effector cells or immunomodulators in the immune response to helminth infection? Parasite Immunol. 2010;32: 1–19. doi: 10.1111/j.1365-3024.2009.01147.x 20042003

[48] Bonne-Année S, Hess JA, Abraham D. Innate and adaptive immunity to the nematode *Strongyloides stercoralis* in a mouse model. Immunol Res. 2011;51: 205–214. doi: 10.1007/s12026-011-8258-2 22101674 PMC6707741

[49] Masure D, Vlaminck J, Wang T, Chiers K, Van den Broeck W, Vercruysse J, et al. A role for eosinophils in the intestinal immunity against infective *Ascaris suum* larvae. PLoS Negl Trop Dis. 2013;7: e2138. doi: 10.1371/journal.pntd.0002138 23556022 PMC3605247

[50] Weatherhead JE, Gazzinelli-Guimaraes P, Knight JM, Fujiwara R, Hotez PJ, Bottazzi ME, et al. Host Immunity and Inflammation to Pulmonary Helminth Infections. Front Immunol. 2020;11: 594520. doi: 10.3389/fimmu.2020.594520 33193446 PMC7606285

[51] Chen F, Liu Z, Wu W, Rozo C, Bowdridge S, Millman A, et al. An essential role for the Th2-type response in limiting tissue damage during helminth infection. Nat Med. 2012;18: 260–266. doi: 10.1038/nm.2628 22245779 PMC3274634

[52] Bonne-Année S, Kerepesi LA, Hess JA, O’Connell AE, Lok JB, Nolan TJ, et al. Human and mouse macrophages collaborate with neutrophils to kill larval *Strongyloides stercoralis*. Infect Immun. 2013;81: 3346–3355. doi: 10.1128/IAI.00625-13 23798541 PMC3754234

[53] Sutherland TE, Logan N, Rückerl D, Humbles AA, Allan SM, Papayannopoulos V, et al. Chitinase-like proteins promote IL-17-mediated neutrophilia in a trade-off between nematode killing and host damage. Nat Immunol. 2014;15: 1116–1125. doi: 10.1038/ni.3023 25326751 PMC4338525

[54] Egholm C, Heeb LEM, Impellizzieri D, Boyman O. The Regulatory Effects of Interleukin-4 Receptor Signaling on Neutrophils in Type 2 Immune Responses. Front Immunol. 2019;10: 2507. doi: 10.3389/fimmu.2019.02507 31708926 PMC6821784

[55] Cooper PJ, Chico ME, Sandoval C, Espinel I, Guevara A, Kennedy MW, et al. Human infection with *Ascaris lumbricoides* is associated with a polarized cytokine response. J Infect Dis. 2000;182: 1207–1213. doi: 10.1086/315830 10979919

[56] Jackson JA, Turner JD, Rentoul L, Faulkner H, Behnke JM, Hoyle M, et al. T helper cell type 2 responsiveness predicts future susceptibility to gastrointestinal nematodes in humans. J Infect Dis. 2004;190: 1804–1811. doi: 10.1086/425014 15499537

[57] Broadhurst MJ, Leung JM, Kashyap V, McCune JM, Mahadevan U, McKerrow JH, et al. IL-22+ CD4+ T cells are associated with therapeutic Trichuris trichiura infection in an ulcerative colitis patient. Sci Transl Med. 2010;2: 60ra88. doi: 10.1126/scitranslmed.3001500 21123809

[58] Dige A, Rasmussen TK, Nejsum P, Hagemann-Madsen R, Williams AR, Agnholt J, et al. Mucosal and systemic immune modulation by *Trichuris trichiura* in a self-infected individual. Parasite Immunol. 2017;39. doi: 10.1111/pim.12394 27743501

[59] Rotman HL, Yutanawiboonchai W, Brigandi RA, Leon O, Gleich GJ, Nolan TJ, et al. *Strongyloides stercoralis*: eosinophil-dependent immune-mediated killing of third stage larvae in BALB/cByJ mice. Exp Parasitol. 1996;82: 267–278. doi: 10.1006/expr.1996.0034 8631378

[60] Herbert DR, Lee JJ, Lee NA, Nolan TJ, Schad GA, Abraham D. Role of IL-5 in innate and adaptive immunity to larval *Strongyloides stercoralis* in mice. J Immunol. 2000;165: 4544–4551. doi: 10.4049/jimmunol.165.8.4544 11035095

[61] Culley FJ, Brown A, Girod N, Pritchard DI, Williams TJ. Innate and cognate mechanisms of pulmonary eosinophilia in helminth infection. Eur J Immunol. 2002;32: 1376–1385. doi: 10.1002/1521-4141(200205)32:5&lt;1376::AID-IMMU1376&gt;3.0.CO;2-8 11981825

[62] Galioto AM, Hess JA, Nolan TJ, Schad GA, Lee JJ, Abraham D. Role of eosinophils and neutrophils in innate and adaptive protective immunity to larval *Strongyloides stercoralis* in mice. Infect Immun. 2006;74: 5730–5738. doi: 10.1128/IAI.01958-05 16988250 PMC1594891

[63] Lalani T, Simmons RK, Ahmed AR. Biology of IL-5 in health and disease. Ann Allergy Asthma Immunol. 1999;82: 317–332; quiz 332–333. doi: 10.1016/S1081-1206(10)63281-4 10227331

[64] Roboz GJ, Rafii S. Interleukin-5 and the regulation of eosinophil production. Curr Opin Hematol. 1999;6: 164–168. doi: 10.1097/00062752-199905000-00007 10226737

[65] Takatsu K, Kouro T, Nagai Y. Interleukin 5 in the link between the innate and acquired immune response. Adv Immunol. 2009;101: 191–236. doi: 10.1016/S0065-2776(08)01006-7 19231596

