# Supplementary figures and images for: Dose-response effects of multiple Ascaris suum exposures and their impact on lung protection during larval ascariasis
Source: PLoS Negl Trop Dis. 2024 Dec 2;18(12):e0012678. doi: 10.1371/journal.pntd.0012678 (PMC11637409; doi:10.1371/journal.pntd.0012678)

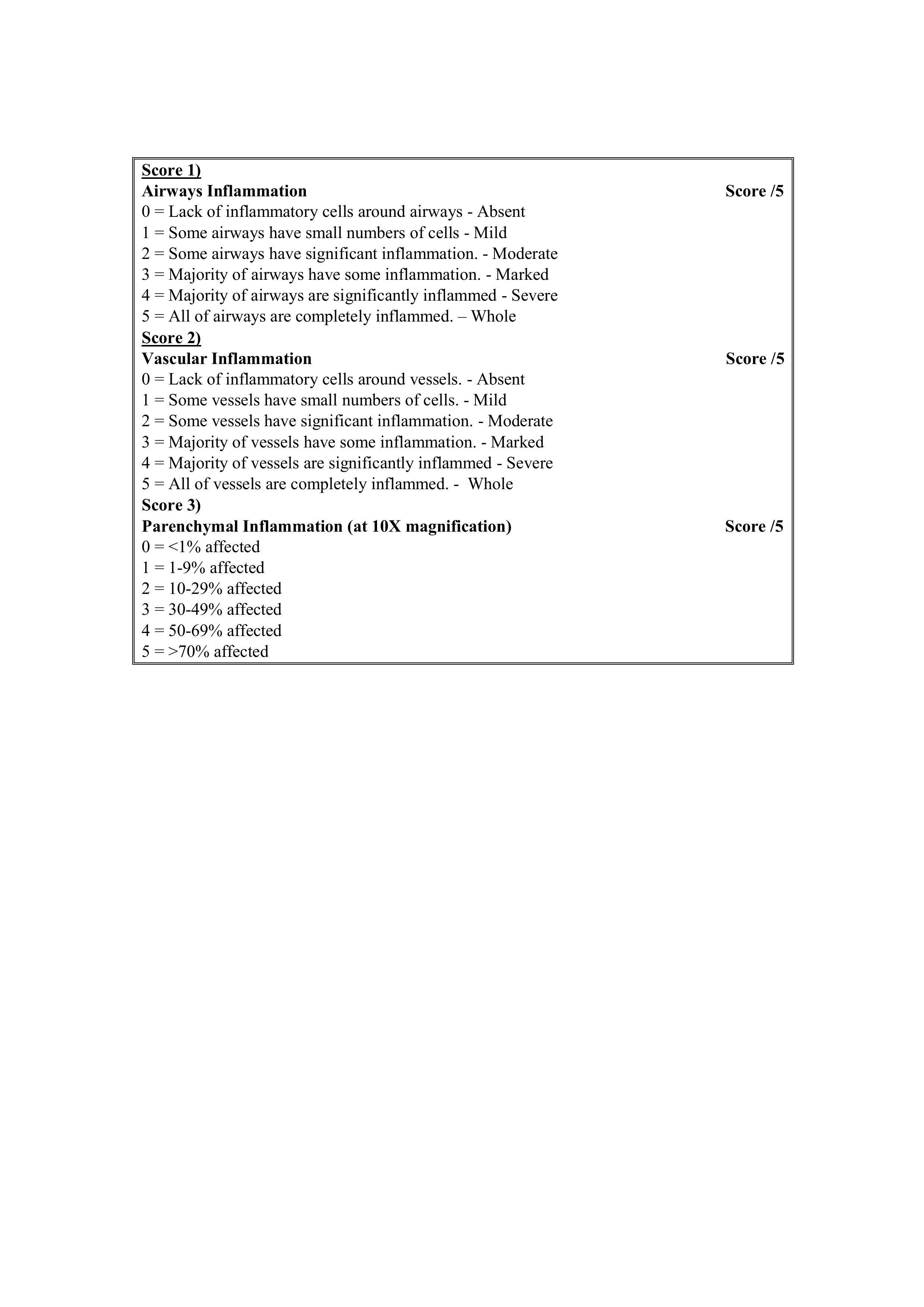

Supplement: S1 Fig — (TIFF) [file pntd.0012678.s001.tiff]
