# Supplementary material for: Dose-response effects of multiple Ascaris suum exposures and their impact on lung protection during larval ascariasis
Source: PLoS Negl Trop Dis. 2024 Dec 2;18(12):e0012678. doi: 10.1371/journal.pntd.0012678 (PMC11637409; doi:10.1371/journal.pntd.0012678)
Supplement: S1 Table — (PDF) [file pntd.0012678.s002.pdf]

Table 1: Analyses and respective comparative p-values

Parasite burden	Comparative Groups	P value
BAL	SI vs. RE 2x	**0,0304**
	SI vs. RE 3x	**0,0003**
	RE 2x vs. RE 3x	0,4331
	SI vs. RE 25	>0,9999
	SI vs. RE 250	**0,0059**
	SI vs. RE 2500	**<0,0001**
	RE 25 vs. RE 250	0,1173
	RE 25 vs. RE 2500	**0,0031**
	RE 250 vs. RE 2500	>0,9999
Lung tissue	SI vs. RE 2x	**0,042**
	SI vs. RE 3x	**0,0008**
	RE 2x vs. RE 3x	0,7722
	SI vs. RE 25	0,9911
	SI vs. RE 250	**0,0002**
	SI vs. RE 2500	**0,0022**
	RE 25 vs. RE 250	**0,0289**
	RE 25 vs. RE 2500	0,1378
	RE 250 vs. RE 2500	>0,9999
Total	SI vs. RE 2x	**0,0449**
	SI vs. RE 3x	**0,0007**
	RE 2x vs. RE 3x	0,708
	SI vs. RE 25	>0,9999
	SI vs. RE 250	**0,0014**
	SI vs. RE 2500	**0,0005**
	RE 25 vs. RE 250	0,0505
	RE 25 vs. RE 2500	**0,0176**
	RE 250 vs. RE 2500	>0,9999

Antibody	Comparative Groups	P value
Ascaris-specific IgG	NI vs. SI	>0,9999
	NI vs. RE 2x	**<0,0001**
	NI vs. RE 3x	**<0,0001**
	SI vs. RE 2x	**<0,0001**
	SI vs. RE 3x	**<0,0001**
	RE 2x vs. RE 3x	**0,0001**
	NI vs. SI	>0,9999
	NI vs. RE 25	**<0,0001**
	NI vs. RE 250	**<0,0001**
	NI vs. RE 2500	**<0,0001**
	SI vs. RE 25	**<0,0001**
	SI vs. RE 250	**<0,0001**
	SI vs. RE 2500	**<0,0001**
	RE 25 vs. RE 250	**0,0012**
	RE 25 vs. RE 2500	**0,0138**
	RE 250 vs. RE 2500	>0,9999
IgG 1	NI vs. SI	>0,9999
	NI vs. RE 2x	**0,0312**
	NI vs. RE 3x	**0,0007**
	SI vs. RE 2x	0,0742
	SI vs. RE 3x	**0,0021**
	RE 2x vs. RE 3x	>0,9999
	NI vs. SI	>0,9999
	NI vs. RE 25	0,1422
	NI vs. RE 250	**0,0019**
	NI vs. RE 2500	**0,0017**
	SI vs. RE 25	0,2662
	SI vs. RE 250	**0,0047**
	SI vs. RE 2500	**0,0043**

Cellular activity	Comparative Groups	P value
EPO	NI vs. SI	0,7287
	NI vs. RE 2x	**<0,0001**
	NI vs. RE 3x	**<0,0001**
	SI vs. RE 2x	**<0,0001**
	SI vs. RE 3x	**<0,0001**
	RE 2x vs. RE 3x	**0,001**
	NI vs. SI	>0,9999
	NI vs. RE 25	**<0,0001**
	NI vs. RE 250	**<0,0001**
	NI vs. RE 2500	**<0,0001**
	SI vs. RE 25	**<0,0001**
	SI vs. RE 250	**<0,0001**
	SI vs. RE 2500	**0,0001**
	RE 25 vs. RE 250	**0,0001**
	RE 25 vs. RE 2500	**<0,0001**
	RE 250 vs. RE 2500	>0,9999
MPO	NI vs. SI	0,5098
	NI vs. RE 2x	**<0,0001**
	NI vs. RE 3x	**0,0068**
	SI vs. RE 2x	**0,0337**
	SI vs. RE 3x	0,6564
	RE 2x vs. RE 3x	>0,9999
	NI vs. SI	>0,9999
	NI vs. RE 25	**<0,0001**
	NI vs. RE 250	**0,0109**
	NI vs. RE 2500	**0,009**
	SI vs. RE 25	**0,0105**
	SI vs. RE 250	0,7193
	SI vs. RE 2500	0,5604
	RE 25 vs. RE 250	>0,9999
	RE 25 vs. RE 2500	>0,9999
	RE 250 vs. RE 2500	>0,9999
NAG	NI vs. SI	>0,9999
	NI vs. RE 2x	**0,001**
	NI vs. RE 3x	**0,003**
	SI vs. RE 2x	**0,0011**
	SI vs. RE 3x	**0,0033**
	RE 2x vs. RE 3x	>0,9999
	NI vs. SI	>0,9999
	NI vs. RE 25	0,2968
	NI vs. RE 250	**0,0035**
	NI vs. RE 2500	**0,0034**
	SI vs. RE 25	**0,3809**
	SI vs. RE 250	**0,0039**
	SI vs. RE 2500	**0,0039**
	RE 25 vs. RE 250	0,994
	RE 25 vs. RE 2500	0,8298
	RE 250 vs. RE 2500	>0,9999

Citokyne levels	Comparative Groups	P value
IL-5	NI vs. SI	>0,9999
	NI vs. RE 2x	**0,0061**
	NI vs. RE 3x	**0,0034**
	SI vs. RE 2x	0,0743
	SI vs. RE 3x	**0,0397**
	RE 2x vs. RE 3x	>0,9999
	NI vs. SI	>0,9999
	NI vs. RE 25	0,147
	NI vs. RE 250	**0,0261**

	RE 25 vs. RE 250	>0,9999
	RE 25 vs. RE 2500	>0,9999
	RE 250 vs. RE 2500	>0,9999
IgG 2a	NI vs. SI	>0,9999
	NI vs. RE 2x	**<0,0001**
	NI vs. RE 3x	**<0,0001**
	SI vs. RE 2x	**0,0005**
	SI vs. RE 3x	**<0,0001**
	RE 2x vs. RE 3x	0,7386
	NI vs. SI	0,4703
	NI vs. RE 25	0,4296
	NI vs. RE 250	**0,0029**
	NI vs. RE 2500	**<0,0001**
	SI vs. RE 25	>0,9999
	SI vs. RE 250	>0,9999
	SI vs. RE 2500	0,0788
	RE 25 vs. RE 250	>0,9999
	RE 25 vs. RE 2500	0,1482
	RE 250 vs. RE 2500	>0,9999
IgG 2b	NI vs. SI	0,9619
	NI vs. RE 2x	**0,0005**
	NI vs. RE 3x	**<0,0001**
	SI vs. RE 2x	**0,0185**
	SI vs. RE 3x	**<0,0001**
	RE 2x vs. RE 3x	0,2615
	NI vs. SI	>0,9999
	NI vs. RE 25	>0,9999
	NI vs. RE 250	**0,0023**
	NI vs. RE 2500	**<0,0001**
	SI vs. RE 25	>0,9999
	SI vs. RE 250	0,0845
	SI vs. RE 2500	**0,0002**
	RE 25 vs. RE 250	**0,0344**
	RE 25 vs. RE 2500	**<0,0001**
	RE 250 vs. RE 2500	0,2904
IgG 3	NI vs. SI	>0,9999
	NI vs. RE 2x	**<0,0001**
	NI vs. RE 3x	**<0,0001**
	SI vs. RE 2x	**<0,0001**
	SI vs. RE 3x	**<0,0001**
	RE 2x vs. RE 3x	**0,0428**
	NI vs. SI	>0,9999
	NI vs. RE 25	0,5045
	NI vs. RE 250	**0,0039**
	NI vs. RE 2500	**0,0001**
	SI vs. RE 25	>0,9999
	SI vs. RE 250	0,0722
	SI vs. RE 2500	**0,0052**
	RE 25 vs. RE 250	>0,9999
	RE 25 vs. RE 2500	0,1762
	RE 250 vs. RE 2500	>0,9999
SIgA	NI vs. SI	>0,9999
	NI vs. RE 2x	**0,0147**
	NI vs. RE 3x	**0,0006**
	SI vs. RE 2x	0,1009
	SI vs. RE 3x	**0,0063**
	RE 2x vs. RE 3x	>0,9999
	NI vs. SI	>0,9999
	NI vs. RE 25	0,1819
	NI vs. RE 2500	**0,0053**
	SI vs. RE 25	>0,9999
	SI vs. RE 250	0,3116
	SI vs. RE 2500	0,0687
	RE 25 vs. RE 250	>0,9999
	RE 25 vs. RE 2500	>0,9999
	RE 250 vs. RE 2500	>0,9999
IL-13	NI vs. SI	0,6333
	NI vs. RE 2x	**0,0016**
	NI vs. RE 3x	**0,0008**
	SI vs. RE 2x	**<0,0001**
	SI vs. RE 3x	**<0,0001**
	RE 2x vs. RE 3x	>0,9999
	NI vs. SI	0,6088
	NI vs. RE 25	>0,9999
	NI vs. RE 250	**0,0083**
	NI vs. RE 2500	**0,0001**
	SI vs. RE 25	**0,0154**
	SI vs. RE 250	**<0,0001**
	SI vs. RE 2500	**<0,0001**
	RE 25 vs. RE 250	0,418
	RE 25 vs. RE 2500	**0,0071**
	RE 250 vs. RE 2500	0,768
IFN-γ	NI vs. SI	0,9476
	NI vs. RE 2x	0,1278
	NI vs. RE 3x	0,4653
	SI vs. RE 2x	**0,001**
	SI vs. RE 3x	**0,0089**
	RE 2x vs. RE 3x	>0,9999
	NI vs. SI	0,2816
	NI vs. RE 25	>0,9999
	NI vs. RE 250	0,0755
	NI vs. RE 2500	0,25
	SI vs. RE 25	>0,9999
	SI vs. RE 250	**0,0001**
	SI vs. RE 2500	**0,0007**
	RE 25 vs. RE 250	**0,0014**
	RE 25 vs. RE 2500	**0,0062**
	RE 250 vs. RE 2500	>0,9999
TNF-α	NI vs. SI	0,2133
	NI vs. RE 2x	**0,0463**
	NI vs. RE 3x	**0,0004**
	SI vs. RE 2x	**0,0002**
	SI vs. RE 3x	**<0,0001**
	RE 2x vs. RE 3x	0,2148
	NI vs. SI	0,2278
	NI vs. RE 25	>0,9999
	NI vs. RE 250	**0,0184**
	NI vs. RE 2500	**0,0001**
	SI vs. RE 25	0,1426
	SI vs. RE 250	**<0,0001**
	SI vs. RE 2500	**<0,0001**
	RE 25 vs. RE 250	**0,0315**
	RE 25 vs. RE 2500	**0,0002**
	RE 250 vs. RE 2500	0,4343

**Histopathological Score**	**Comparative Groups**	**P value**
Total Inflammation	NI vs. SI	0,2723
	NI vs. RE 2x	**0,0003**
	NI vs. RE 3x	**0,003**

	NI vs. RE 250	**0,0054**
	NI vs. RE 2500	**<0,0001**
	SI vs. RE 25	0,6405
	SI vs. RE 250	**0,0316**
	SI vs. RE 2500	**0,0006**
	RE 25 vs. RE 250	>0,9999
	RE 25 vs. RE 2500	0,246
	RE 250 vs. RE 2500	>0,9999

**Leukocyte recruitment**	**Comparative Groups**	**P value**
Total Leukocytes	NI vs. SI	0,9517
	NI vs. RE 2x	**<0,0001**
	NI vs. RE 3x	**<0,0001**
	SI vs. RE 2x	**<0,0001**
	SI vs. RE 3x	**<0,0001**
	RE 2x vs. RE 3x	>0,9999
	NI vs. SI	0,6449
	NI vs. RE 25	**0,0007**
	NI vs. RE 250	**<0,0001**
	NI vs. RE 2500	**<0,0001**
	SI vs. RE 25	0,1353
	SI vs. RE 250	**0,0009**
	SI vs. RE 2500	**<0,0001**
	RE 25 vs. RE 250	0,7129
	RE 25 vs. RE 2500	**<0,0001**
	RE 250 vs. RE 2500	**<0,0001**
Eosinophils	NI vs. SI	0,306
	NI vs. RE 2x	**0,0053**
	NI vs. RE 3x	**0,0002**
	SI vs. RE 2x	0,7356
	SI vs. RE 3x	0,1052
	RE 2x vs. RE 3x	>0,9999
	NI vs. SI	>0,9999
	NI vs. RE 25	0,0523
	NI vs. RE 250	**0,001**
	NI vs. RE 2500	**<0,0001**
	SI vs. RE 25	>0,9999
	SI vs. RE 250	0,1272
	SI vs. RE 2500	**0,0066**
	RE 25 vs. RE 250	>0,9999
	RE 25 vs. RE 2500	0,4951
	RE 250 vs. RE 2500	>0,9999
Neutrophils	NI vs. SI	0,3058
	NI vs. RE 2x	**0,0014**
	NI vs. RE 3x	**0,005**
	SI vs. RE 2x	0,3059
	SI vs. RE 3x	0,6593
	RE 2x vs. RE 3x	>0,9999
	NI vs. SI	0,213
	NI vs. RE 25	0,0572
	NI vs. RE 250	**0,0288**
	NI vs. RE 2500	**0,0004**
	SI vs. RE 25	>0,9999
	SI vs. RE 250	>0,9999
	SI vs. RE 2500	0,3773
	RE 25 vs. RE 250	>0,9999
	RE 25 vs. RE 2500	>0,9999
	RE 250 vs. RE 2500	>0,9999
Macrophages	NI vs. SI	>0,9999
	NI vs. RE 2x	**<0,0001**
	SI vs. RE 2x	0,2411
	SI vs. RE 3x	0,7183
	RE 2x vs. RE 3x	>0,9999
	NI vs. SI	0,144
	NI vs. RE 25	**0,0005**
	NI vs. RE 250	0,1677
	NI vs. RE 2500	**0,001**
	SI vs. RE 25	0,8893
	SI vs. RE 250	>0,9999
	SI vs. RE 2500	>0,9999
	RE 25 vs. RE 250	0,7934
	RE 25 vs. RE 2500	>0,9999
	RE 250 vs. RE 2500	>0,9999
Peribronchial Inflammation	NI vs. SI	0,1444
	NI vs. RE 2x	**0,0004**
	NI vs. RE 3x	**0,0022**
	SI vs. RE 2x	0,4819
	SI vs. RE 3x	0,9687
	RE 2x vs. RE 3x	>0,9999
	NI vs. SI	0,0733
	NI vs. RE 25	**0,0003**
	NI vs. RE 250	0,1369
	NI vs. RE 2500	**0,0009**
	SI vs. RE 25	>0,9999
	SI vs. RE 250	>0,9999
	SI vs. RE 2500	>0,9999
	RE 25 vs. RE 250	0,7177
	RE 25 vs. RE 2500	>0,9999
	RE 250 vs. RE 2500	>0,9999
Perivascular Inflammation	NI vs. SI	0,4914
	NI vs. RE 2x	**0,0003**
	NI vs. RE 3x	**0,0031**
	SI vs. RE 2x	0,1131
	SI vs. RE 3x	0,4315
	RE 2x vs. RE 3x	>0,9999
	NI vs. SI	0,3847
	NI vs. RE 25	**0,0041**
	NI vs. RE 250	0,1503
	NI vs. RE 2500	**0,001**
	SI vs. RE 25	>0,9999
	SI vs. RE 250	>0,9999
	SI vs. RE 2500	0,5736
	RE 25 vs. RE 250	>0,9999
	RE 25 vs. RE 2500	>0,9999
	RE 250 vs. RE 2500	>0,9999
Parenchymal Inflammation	NI vs. SI	>0,9999
	NI vs. RE 2x	**0,0076**
	NI vs. RE 3x	**0,0198**
	SI vs. RE 2x	0,0801
	SI vs. RE 3x	0,1584
	RE 2x vs. RE 3x	>0,9999
	NI vs. SI	>0,9999
	NI vs. RE 25	0,1635
	NI vs. RE 250	**0,041**
	NI vs. RE 2500	**0,0048**
	SI vs. RE 25	>0,9999
	SI vs. RE 250	0,4114
	SI vs. RE 2500	0,0706
	RE 25 vs. RE 250	>0,9999
	RE 25 vs. RE 2500	>0,9999

	NI vs. RE 3x	**0,0007**
	SI vs. RE 2x	**<0,0001**
	SI vs. RE 3x	**0,0089**
	RE 2x vs. RE 3x	**0,0441**
	NI vs. SI	0,5444
	NI vs. RE 25	**0,0265**
	NI vs. RE 250	0,0621
	NI vs. RE 2500	**0,0001**
	SI vs. RE 25	>0,9999
	SI vs. RE 250	>0,9999
	SI vs. RE 2500	0,066
	RE 25 vs. RE 250	>0,9999
	RE 25 vs. RE 2500	>0,9999
	RE 250 vs. RE 2500	0,6476
<u>Lymphocytes</u>	NI vs. SI	>0,9999
	NI vs. RE 2x	**0,0185**
	NI vs. RE 3x	**<0,0001**
	SI vs. RE 2x	**0,0324**
	SI vs. RE 3x	**<0,0001**
	RE 2x vs. RE 3x	0,1247
	NI vs. SI	>0,9999
	NI vs. RE 25	0,9641
	NI vs. RE 250	**0,0029**
	NI vs. RE 2500	**<0,0001**
	SI vs. RE 25	>0,9999
	SI vs. RE 250	**0,0059**
	SI vs. RE 2500	**<0,0001**
	RE 25 vs. RE 250	0,3224
	RE 25 vs. RE 2500	**0,0005**
	RE 250 vs. RE 2500	0,0864

	**Comparative Groups**	**P value**
<u>Hemoglobin</u>	NI vs. SI	**<0,0001**
	NI vs. RE 2x	0,3412
	NI vs. RE 3x	>0,9999
	SI vs. RE 2x	**0,0015**
	SI vs. RE 3x	**0,0006**
	RE 2x vs. RE 3x	>0,9999
	NI vs. SI	**0,0016**
	NI vs. RE 25	0,057
	NI vs. RE 250	>0,9999
	NI vs. RE 2500	>0,9999
	SI vs. RE 25	>0,9999
	SI vs. RE 250	**0,007**
	SI vs. RE 2500	0,556
	RE 25 vs. RE 250	0,1948
	RE 25 vs. RE 2500	>0,9999
	RE 250 vs. RE 2500	>0,9999
<u>Total protein</u>	NI vs. SI	**0,0007**
	NI vs. RE 2x	0,0642
	NI vs. RE 3x	0,1982
	SI vs. RE 2x	0,9402
	SI vs. RE 3x	0,505
	RE 2x vs. RE 3x	>0,9999
	NI vs. SI	**0,002**
	NI vs. RE 25	**0,0029**
	NI vs. RE 250	>0,9999
	NI vs. RE 2500	0,1474
	SI vs. RE 25	>0,9999
	SI vs. RE 250	0,2094
	SI vs. RE 2500	>0,9999

	RE 250 vs. RE 2500	>0,9999
Hemorrhage	NI vs. SI	**0,0001**
	NI vs. RE 2x	0,0876
	NI vs. RE 3x	>0,9999
	SI vs. RE 2x	0,4353
	SI vs. RE 3x	**0,0251**
	RE 2x vs. RE 3x	>0,9999
	NI vs. SI	**0,0017**
	NI vs. RE 25	**0,0016**
	NI vs. RE 250	>0,9999
	NI vs. RE 2500	>0,9999
	SI vs. RE 25	>0,9999
	SI vs. RE 250	0,1027
	SI vs. RE 2500	0,1011
	RE 25 vs. RE 250	0,0875
	RE 25 vs. RE 2500	0,0857
	RE 250 vs. RE 2500	>0,9999

**Physiological pulmonary**	**Comparative Groups**	**P value**
<u>Pulmonary Resistance</u>	NI vs. SI	**0,0066**
	NI vs. RE 2x	0,0539
	NI vs. RE 3x	0,1863
	SI vs. RE 2x	>0,9999
	SI vs. RE 3x	>0,9999
	RE 2x vs. RE 3x	>0,9999
	NI vs. SI	**0,0047**
	NI vs. RE 25	0,0717
	NI vs. RE 250	0,1054
	NI vs. RE 2500	0,2399
	SI vs. RE 25	>0,9999
	SI vs. RE 250	>0,9999
	SI vs. RE 2500	>0,9999
	RE 25 vs. RE 250	>0,9999
	RE 25 vs. RE 2500	>0,9999
	RE 250 vs. RE 2500	>0,9999
<u>Dynamic Compliance</u>	NI vs. SI	**0,0002**
	NI vs. RE 2x	**0,001**
	NI vs. RE 3x	**0,0403**
	SI vs. RE 2x	>0,9999
	SI vs. RE 3x	0,4424
	RE 2x vs. RE 3x	0,7811
	NI vs. SI	**0,0003**
	NI vs. RE 25	**0,0021**
	NI vs. RE 250	0,0971
	NI vs. RE 2500	0,0675
	SI vs. RE 25	>0,9999
	SI vs. RE 250	0,3393
	SI vs. RE 2500	0,7791
	RE 25 vs. RE 250	>0,9999
	RE 25 vs. RE 2500	>0,9999
	RE 250 vs. RE 2500	>0,9999

	RE 25 vs. RE 250	0,2366
	RE 25 vs. RE 2500	>0,9999
	RE 250 vs. RE 2500	>0,9999
